# Characterising Biosecurity Initiatives Globally to Support the Development of a Progressive Management Pathway for Terrestrial Animals: A Scoping Review

**DOI:** 10.3390/ani13162672

**Published:** 2023-08-19

**Authors:** Nina Militzer, Melissa McLaws, Andriy Rozstalnyy, Yushan Li, Madhur Dhingra, Aashima Auplish, Koen Mintiens, Mirzet Sabirovic, Sophie von Dobschuetz, Martin Heilmann

**Affiliations:** 1 Food and Agriculture Organization of United Nations (FAO), 00153 Rome, Italy; 2 Deutsche Gesellschaft für Internationale Zusammenarbeit (GIZ) GmbH, 53113 Bonn, Germany

**Keywords:** biosecurity, progressive management pathway, livestock, One Health, value chain, SWOT analysis, recommendations

## Abstract

**Simple Summary:**

Biosecurity is a strategic and integrated approach to analysing and managing risks to human, animal, and plant life and health, and associated environmental risks to the environment. Despite its growing importance worldwide, not least because of the COVID-19 pandemic, elements and principles needed to successfully expand biosecurity from the local (e.g., enterprises) to the global (e.g., border) level have not been identified. To gain more insights on the current status of biosecurity-relevant literature and elements and principles, a review was performed, including the identification of strengths, weaknesses, opportunities, and threats of existing biosecurity initiatives, projects, and programs. Initial results from this review were complemented with a stakeholder survey. Key results include that most records focus on high-income countries, traditional livestock species (pigs, poultry, large ruminants), viral hazards, and biosecurity at the production level. The findings also highlight the need for initiatives such as the Progressive Management Pathway for Terrestrial Animal Biosecurity (PMP-TAB), currently being designed by the Food and Agriculture Organization (FAO), to build sustainable biosecurity systems globally. Based on the findings, key elements and recommendations were identified to support the development of progressive pathway approaches for better biosecurity from a One Health perspective.

**Abstract:**

While biosecurity is of increasing importance globally, there is still limited evidence of the factors or elements that support the progressive and sustainable scaling up of biosecurity along the value chains from the local to the global level. To gain insight into the current body of literature on biosecurity, a mixed-methods approach was used based on a scoping literature review and an online survey with subject matter experts. Six databases were searched for published literature, and textual information from titles and abstracts of all included records (*n* = 266) were analysed through inductive content analysis to build biosecurity-relevant categories and identify strengths, weaknesses, opportunities, and threats (SWOT) of existing biosecurity systems or initiatives (such as projects or programs). Most records focused on initiatives in high-income countries, traditional livestock species (pigs, poultry, and large ruminants), and the production stage and had a disease-specific focus. No records described a comprehensive or global framework to progressively scale up biosecurity. Overall, the findings highlight the need for initiatives such as the FAO Progressive Management Pathway for Terrestrial Animal Biosecurity (FAO-PMP-TAB), which is a stepwise approach for strengthening biosecurity management along value chains to enhance the health, resilience, and sustainability of animal sectors. The findings highlight important elements and provide recommendations useful for developing approaches or a global framework to progressively improve biosecurity management.

## 1. Introduction

The Food and Agriculture Organization of the United Nations (FAO) defines biosecurity as a strategic and integrated approach to analysing and managing relevant risks to human, animal, and plant life and health, and associated risks to the environment [1]. This approach is in line with the integrated and unifying approach of One Health towards the better health of animals, people, and ecosystems [2,3].

The importance of strong biosecurity systems has increased significantly over recent decades with globalisation, intensification of animal production systems, and concomitant increasing trade in food, plant, and animal products and international travel driving the spread of emerging or re-emerging diseases [2,4]. As this is not only limited to infectious diseases, biosecurity covers also other hazards, for instance, residues or pests, and was also associated to antimicrobial usage (AMU) and antimicrobial resistances (AMRs) [4,5]. Beyond the impacts on public health, biosecurity plays a major role in reducing the economic costs of outbreaks at farms [6,7] and on national levels [8]. However, biosecurity goes beyond trade and transnational movements of goods; it builds the “foundation” [9] of disease prevention and control. This has practical implications for communities at the local level and in fast-changing and growing agri-food systems, where the production, processing, and distribution of food, plants, and animals are highly dynamic. However, despite these developments, it is still not well understood how biosecurity can be progressively scaled up from the local (e.g., enterprises) to the global (e.g., border) level, such that successful local initiatives are scaled up, at first nationally and, as appropriate, adapted and replicated in other countries. This would result in improved biosecurity globally.

To that end, FAO is developing a Progressive Management Pathway for Terrestrial Animal Biosecurity (PMP-TAB) [10] to guide countries to develop sustainable biosecurity for healthy and resilient terrestrial animal populations through a collaborative, stepwise approach. This is in line with the application based on One Health principles [11,12]. The PMP-TAB is at the heart of FAO’s Strategic Framework’s aspiration of “Better Production” and the related One Health Programme Priority Area (OHPPA) constituting a globally led, integrated, and coordinated One Health approach to preventing, containing, and addressing the rising losses to agricultural production and adverse ecosystem effects caused by the spread of animal, forestry, plant, and aquatic pests and diseases, including zoonotic infections of pandemic potential and AMR [13].

The objective of this review is to identify, characterise, and analyse (through a SWOT analysis) existing biosecurity-related literature. Further, biosecurity initiatives at the local-to-global levels are captured to have an evidence basis used for the development of the PMP-TAB.

Research questions to be answered are as follows:

- What is the body of literature related to biosecurity that currently exists, and how is it distributed over time and space?
- What are the aspects that are important for implementing sustainable biosecurity systems?
- What are the strengths, weaknesses, opportunities, and threats of existing biosecurity systems or initiatives (such as programs or projects)?
- How can existing evidence support countries in progressively improving biosecurity along the value chain, through the application of One Health principles?

The findings of this review will provide recommendations to consider when developing approaches to progressively improve biosecurity [10].

## 2. Materials and Methods

### 2.1. Literature Search Strategy

This review utilised a mixed-methods approach and included a scoping literature review and a survey with subject matter experts (SMEs). A literature review was conducted to identify and critically analyse the existing body of literature related to the implementation of biosecurity at the enterprise, community, and national levels, to ultimately provide recommendations for the development of the PMP-TAB [14].

Here, the five steps for scoping reviews were conducted as follows: (1) identification of key research questions, (2) identification of relevant studies, (3) selection of studies, (4) data charting/extraction, (5) collating, summarising, and reporting the results [15]. The PRISMA checklist for Scoping Reviews was followed [16]. The review team consisted of one person (N.M.), who developed the protocol, including the research questions, search strategy, eligibility criteria, and data characteristics/classifications, and conducted screening and data extraction in close consultation with other members of the research team (M.M., A.R., M.H., and S.v.D.). Searches were run on 18 April 2022, using the following databases:

- https://pubmed.ncbi.nlm.nih.gov/ (accessed on 18 April 2022)
- https://www.webofscience.com/wos/woscc/basic-search (accessed on 18 April 2022)
- https://www.fao.org/publications/search/en/ (accessed on 18 April 2022)
- https://scholar.google.de/ (accessed on 18 April 2022)
- https://www.ilri.org/publications (accessed on 18 April 2022)
- https://www.worldbank.org/en/research (accessed on 18 April 2022)

The initial search terms were in English, but any record, independent of language, was included following the initial keyword search. No restrictions on the date of publication were applied for the search. No limitation to grey literature nor to specific time periods were set. Searches were limited to title or title and abstract (see Tables S1 and S2 for the detailed search strategy and search terms used). If no abstract was available, the screening focused on any available text section (e.g., summary, presentation, introduction, factsheet, highlights, press articles, or website).

After the initial search, it was performed and followed an extraction into EndNote (Version 20). After removal of duplicates, records were transferred into Rayyan (Rayyan Systems Inc. Cambridge, MA, USA) [17], followed by an initial round of screening of titles and abstracts for eligibility criteria (see Table S3 for the inclusion and exclusion criteria). Eligibility criteria were based on the population, intervention, comparison, outcome (PICO) checklist. The focus was on biosecurity systems, initiatives, and programs, but also on applied tools to improve or assess biosecurity. Nevertheless, records were excluded if there was only a focus on (farm) biosecurity measurements, just an assessment of (farm) biosecurity status, or a strong focus on only risk/hazard/diseases. Further, we omitted records referring to laboratory biosecurity and biosafety, biological weapons, and/or bioterrorism. Records with this focus were, however, included if the text refers to a certain system, initiative, programs, case, or tool. This was the same also for behaviour science/social science-related biosecurity records. While infectious diseases and biological hazards are in the focus of this review, it also considered chemical and physical hazards.

**Figure 1:**
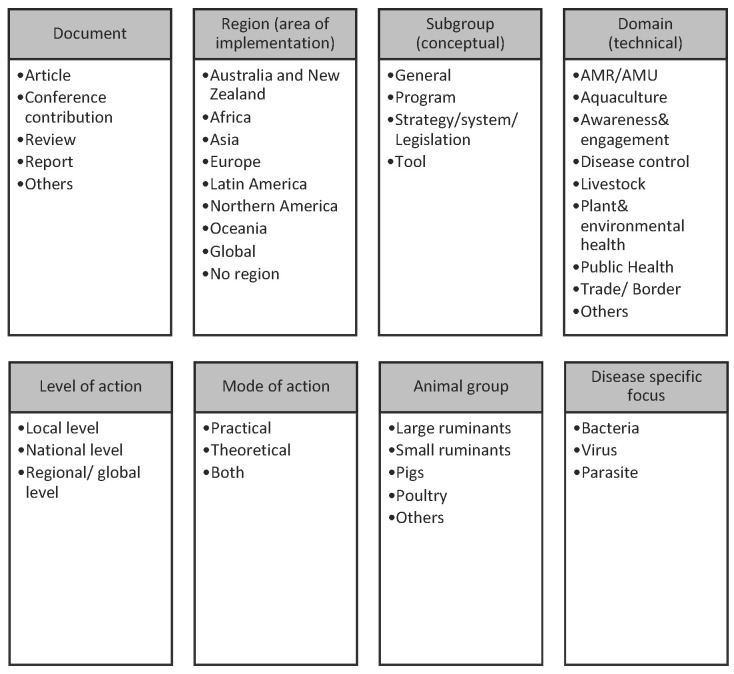
Overview of categories built from the textual information in identified records. More details are available in Tables S4 and S5.

Records were excluded if no title or abstract (or similar text) was available online. Any uncertainties around the inclusion of records were resolved during weekly meetings among authors (N.M., M.M., A.R., and M.H.), specifically by an author who had not already screened the record in question. Included records were exported to Microsoft^®^ Office Excel, Version 16, where data extraction was carried out. Results were analysed descriptively by spatio-temporal occurrence (by year and region). Regions were categorised based on the United Nations geoscheme M49 (https://unstats.un.org/unsd/methodology/m49/ accessed on 12 June 2023), with certain modifications: “Africa”, “Asia”, “Europe”, “Australia and New Zealand” (incl. Tokelau), “Oceania” (incl. Melanesia, Micronesia, Polynesia), “Latin America” (incl. Caribbean, Central and South America), “Northern America”, “Global”, and “No region”. When records covered multiple countries, they were counted multiple times in order to ensure comprehensive coverage. Based on the textual data from titles and abstracts, the coding of “categories” was conducted through an inductive content analysis approach [18] including the following steps: (a) drafting research questions, (b) development of inductive categories from record abstracts/texts during screening, (c) the revision of categories, and (d) results interpretation. Figure 1 presents an overview of the main categories that were defined based on the textual information extracted from each record. If applicable, countries were grouped into regions based on their area/location of implementation or reference. Included records were then categorised based on the conceptual nature of the record in regard to its purpose (“subgroup”) and on the technical nature of the record in regard to its field of study (“domain”). Only one categorisation for each category (e.g., region, subgroup, domain, etc.) was performed for each record. Since one objective of this review was to provide an evidence base for the development of the PMP-TAB, we specifically analysed the different records by their scale of implementation (e.g., local, national, regional, or global implementation) and assessed whether initiatives focused on smallholders (e.g., small-scale agriculture). Thus, a smallholder focus was identified through the content analysis (e.g., by keywords, such as small-scale, small-holder, backyard). Further, a scanning on animal post-production stages was also performed based on keywords, such as post-production, slaughter*, process*, retail*, butcher*, consum*.

Each record was also analysed for strengths, weaknesses, opportunities, and threats (SWOT). The SWOT analysis has its origin in management analysis tools [19], but is now a commonly used tool also for the analysis of systems approaches or governance [20]. The SWOT analysis has been performed for the following subgroups: “general”, “program”, and “strategy/system”. The SWOT classifications were identified through direct description of the SWOT by the author in the original text (e.g., “[…] the programme was proven to be highly effective as a means of […]” [21], or “[…] can offer advantages […] [22], or “[…] there are still gaps […]” [23]) or by subjective categorisation based on description (e.g., “[…] which makes the application of this tool […] faster […]” [24] as a strength or “[…] adoption of the […] biosecurity practices is generally low […]” [25] as a weakness).

### 2.2. Biosecurity Survey

An online survey was conducted with SMEs to identify other unpublished, but relevant, biosecurity initiatives; gain further insight into essential components of strong and sustainable biosecurity systems that have been implemented, and triangulate the literature review findings. The survey was administered through the SurveyMonkey platform (Momentive Europe UC, Dublin, Ireland) between 23 May and 8 June 2022. It was shared through an internal FAO mailing list, five FAO regional offices, and eleven sub-regional officers for further distribution at the regional and country level. Additionally, it was distributed through specific FAO networks, such as the AMR technical working group, the livestock technical network, and the One Health network. In total, the networks included about 500 SMEs. Responses to the survey were anonymous. The survey was made available in two languages (English and French) and consisted of four sections with a total of 34 questions (see Table S6).

## 3. Results

### 3.1. Search Results

**Figure 2:**
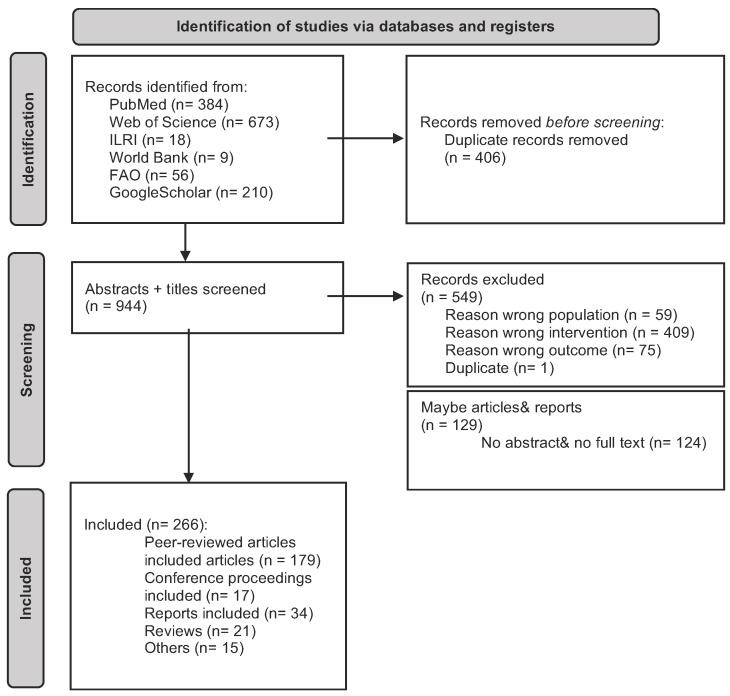
PRISMA flow chart for selection of sources of evidence. Inclusion and exclusion criteria were based on the PICO checklist and are listed in Table S3.

A total of 266 records met the inclusion criteria and were included in the final analysis (Figure 2). A list of all included records can be found in Supplementary File S2.

### 3.2. Spatiotemporal Description of Records

**Figure 3:**
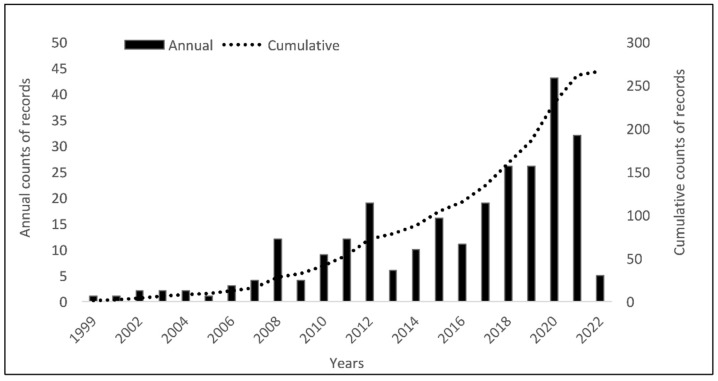
Annual number of records identified in the literature review between 1999 and April 2022.

Records on biosecurity-related topics published between 1999 and 2022 have been increasing since 1999, with peaks in 2008, 2012, and in 2020 (Figure 3). Overall, 54 different countries were referenced, across 7 different regions.

Australia and New Zealand were the most frequently referenced countries (38% of records, *n* = 94), followed by the pre-defined regions Europe (20%), Asia (16%), Northern America (10%), Africa (9%), Oceania (3%), Latin America (2%), and global (2%). To investigate further, we grouped countries by income group, as per the World Bank classification [26]: low-income economies (LICs), lower-middle-income economies (LMICs), upper-middle-income economies (UMICs), and high-income economies (HICs). Most records (71%) described biosecurity-related initiatives in HICs, while 21% related to LMICs and LICs. Of the countries included in the records, 4 countries (Ethiopia, Madagascar, Mozambique, and Uganda) were identified in the LICs, while 19 countries were identified in HICs (including Australia as the most often identified HIC, *n* = 63), 12 countries in UMICs (including China as the most often identified UMICs, *n* = 5), and 19 countries in LMICs (including Vietnam as the most often identified LMIC, *n* = 5).

### 3.3. Classification of Subgroups

**Figure 4:**
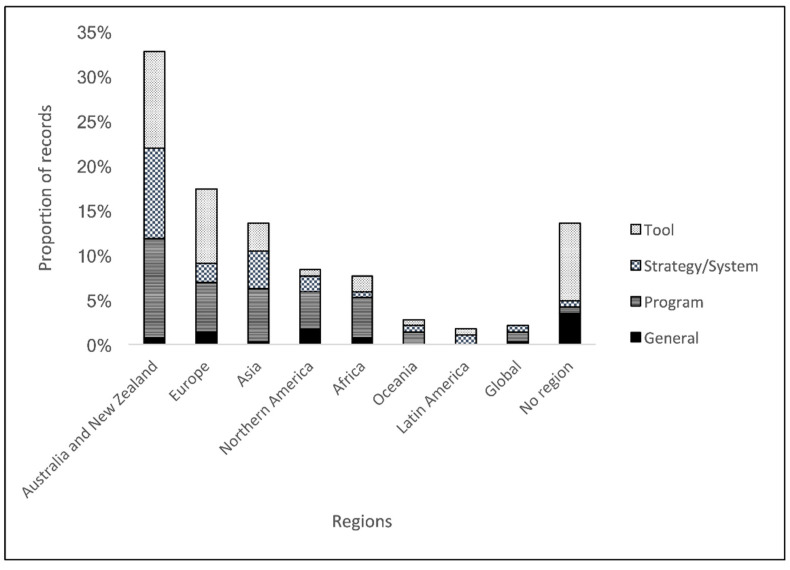
Proportion of records by subgroup and region (*n* = 227/266). Publications referring to more than one region or country (*n* = 11) were respected. Remaining records (*n* = 39) referred to no specific region.

Regarding subgroups, most records referred to biosecurity-related “tools” (35%) and “programs” (34%). Tools included records that referred to diagnostic, technological, or assessment tools (e.g., using the Biocheck.UGent scoring system [27]). Programs, on the other hand, referred to specific projects and/or programs that involve implementation of biosecurity-related activities on the ground (e.g., African Swine Fever control project in Papua New Guinea [28]). “Strategy and systems” were also commonly referred to (22%) and include policy-related documents, for instance, national strategies, plans, legislation, or frameworks (e.g., on biosecurity systems in New Zealand [29]). Finally, “general” records (9%) included overarching topics without a specific focus on the existing subgroups (e.g., on economic aspects [30]). Additional information about the classification can be found in Figure 1 and Supplementary File S1. When subgroups were categorised by region, most records related to the region: “Australia and New Zealand” referred to programs (*n* = 32/94), while most records in Europe included specific tools (*n* = 24/50; Figure 4).

### 3.4. Classification of Domains

The domains refer to the technical focus of the record (Table 1).

**Figure 5:**
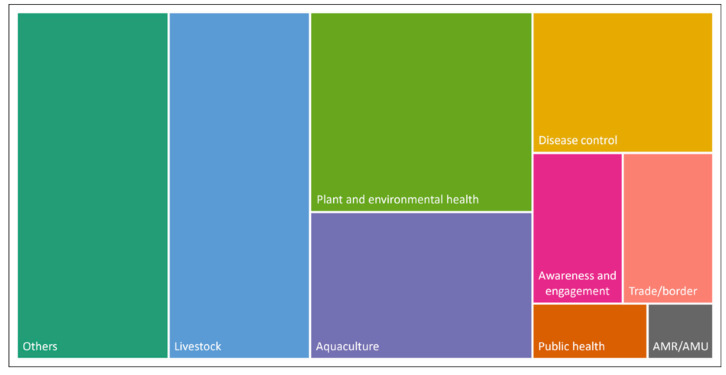
Overview of domains captured in this review. “AMR/AMU” = Antimicrobial resistance/antimicrobial usage. Additional information on each domain is available in Supple-mentary File S1.

Most records (20%) belong to the domain “livestock”, with a focus on specific animal husbandry, livestock farming, or systems. This was followed by “plant and environmental health” (18%), which included records with a major focus on plants, forestry, or environmental matters. Several records (22%) referred to a diverse range of topics and were thus classified as “other”. They included topics such as legislation, governance, and policy, but also surveillance and detection and/or diagnostic focuses. Figure 5 provides an overview of the domains. Additional detail for each domain is available in Supplementary File S1.

**Figure 6:**
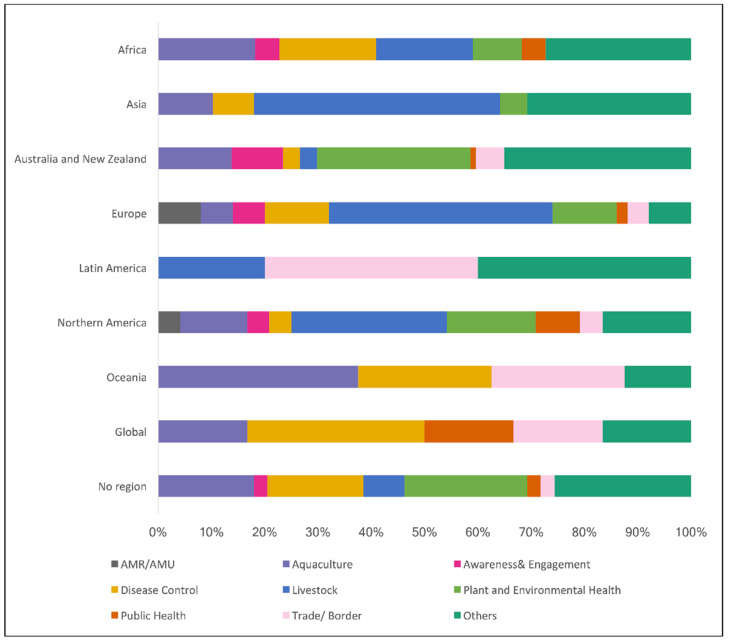
Proportion of domains per each region in percentage (%). Publications referring to more than one region or country were respected.

In terms of the regional distribution of domains, biosecurity initiatives related to ”AMR/AMU” were only recorded in Europe and Northern America, while initiatives related to “aquaculture” were recorded in most regions, with the highest proportion in “Australia and New Zealand” (34%). The domains of “awareness and engagement” (60%), “plant and environmental health” (54%), and “trade/border” (36%) were mainly associated with the region “Australia and New Zealand”. The domain “livestock” occurred most frequently in the region Europe (37%), followed by Asia (32%) (Figure 6).

**Figure 7:**
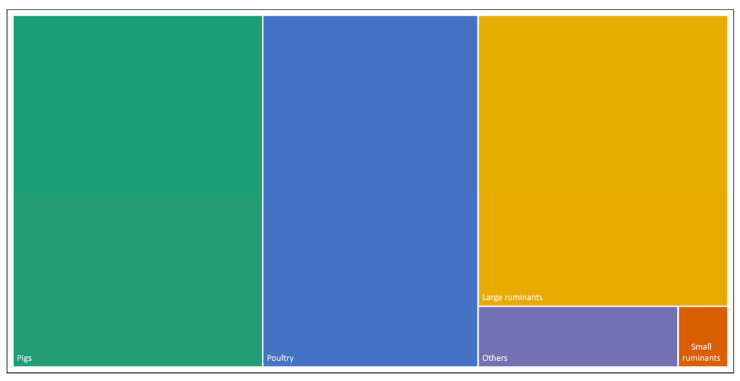
Coverage of specific animal groups by the identified records. The size of each rectangle represents the number of times animal groups were mentioned in the records. “Others” = arthropods, equines, or wildlife.

In terms of animal focus, the domains of “livestock” and “disease control” specifically referred to animal species, including pigs (34%), large ruminants (30%), poultry (30%), and others (6%), including arthropods, horses, or wildlife (Figure 7). In terms of disease focus, most records related to viral (79%) or bacterial diseases (18%). Commonly cited viral diseases included highly pathogenic avian influenza (*n* = 8/29) and African swine fever (*n* = 6/29), while commonly cited bacterial diseases included bovine tuberculosis (*Mycobacterium bovis*, *n* = 3/7). Only one record referred to tick-borne disease (3%).

### 3.5. Classification of Level of Action and Smallholder Focus

Most records referred to biosecurity-related initiatives at the national (43%) or local level (32%), while 6% (*n* = 16/266) referred to initiatives at the regional or global level. Subgroups for local-level initiatives were mostly “programs” (51%) or “tools” (39%), while for the national level, the subgroups were more commonly “strategy/system” (35%), “programs” (31%), or “tools” (26%). Only 15 records focused on smallholders. These records related to ”programs” (93%) or “tools” (7%). The smallholder programs were implemented in Asia (50%) or Africa (30%) and mainly focused on an animal species (80%), specifically, pigs (42%), poultry (33%), or large ruminants (25%). Out of all records, a minority (<10%) referred to biosecurity being implemented at the post-production level (e.g., slaughter, transport, or retail/market level).

### 3.6. SWOTs

A SWOT analysis was conducted to evaluate the identified programs, strategies, and tools related to biosecurity in the included records. Table S5 illustrates the top 10 of each SWOT classification in descending order. The SWOTs are outlined in the following section, with examples of the original keywords identified in the included records and used for the coding presented in parentheses.

The three major strengths were identified, including “multistakeholder” (“partnerships between government and industry”, “relevant stakeholders”, or “collaborative”), “participatory process” (“co-created” or “participatory approach”), and “capacity building” (“training” or “educational”). The major weakness identified was around “compliance” (“low adoption” or “willingness”). Four other weaknesses were identified, including “awareness and perceptions” (“different risk perceptions of stakeholders” or “no awareness”), “time” (“short-term” or “delayed response time”), insufficient or missing “capacity building” (“training” or “professional development”), and “financing and funding” (“costs” or “no […] economic advantages”).

“Upscaling” (“multiplier” or “scaling”) was the most commonly identified opportunity, followed by “law and policy enforcement” (“legislative implementation support” or “recommendations on how these laws may be developed” or “want more, not less, government biosecurity regulation”). In terms of threats, “tourism, mobility” (“high human accessibility” or “travel”), “financing and funding” (“lack of financial resources” or “cost efficiency”), and “demographic change” (“growing population” or “urbanization”) were most frequently identified. As shown in Table S5, some SWOT classifications are represented as strengths, but also as weakness, opportunity, and/or threat. For instance, the classification of “time” is identified as a strength when keywords like “timely”, “rapid”, “long-term”, or “constant” were used in the included records to describe biosecurity initiatives, while the classification of “time” as a weakness was used when keywords like “short-term”, “slow”, or “tensions between […] immediate needs […] and supporting more long-term […]” were used. This is also seen for “knowledge and information sharing”, which is classified as a strength or opportunity when “efficient” or “successful”, and as a weakness or threat when “lack of” or “insufficiency” were used to describe the term.

### 3.7. Survey Results

In total, 21 respondents completed the survey, with 19 completed in English and 2 in French. Forty-four biosecurity initiatives were identified through the survey, which were mostly related to the Asian region (20%). Seventy-two percent of initiatives related to the subgroup “program”. These programs were mostly referring to the national level (30%), followed by “more than one” level (24%), “local” level (20%), or “regional/global” level (13%). Thirteen percent of programs referred to no particular level. The domain of “disease control” (42%) and “others” were the most frequently recorded (42% and 25%, respectively). Others referred to topics on One Health, national strategies, and veterinary training or surveillance programs. Poultry was the dominant animal species recorded (48%), and similar to the literature review, viral diseases were the most common disease-focus of initiatives.

Respondents reported that the essential components and variables for enforcing good biosecurity at the community level and in the private sector were knowledge and capability (of emergency management, biosecurity guidelines, and good practices) and awareness and engagement, including multistakeholder partnerships and cooperation and use of participatory approaches. The most important factor for strengthening biosecurity at the community level was related to feasible, tangible practices. To promote involvement of the private sector, factors like partnerships and power structures (e.g., equality in terms of influence on decisions, access to resources, etc.), awareness, and engagement were highlighted consistently by the respondents. Public bodies were considered as critical players for the effective implementation of biosecurity initiatives. Furthermore, a holistic approach and a wide stakeholder consultation process were frequently mentioned as strengths and opportunities. Surprisingly, challenges on financing were not the most frequently reported weakness or threat. Here, the missing or insufficient capacity building and information sharing and the lack of public sector/government involvement were highlighted as a major weakness or threat (each 26%). In terms of the scope of biosecurity, the respondents reported that biosecurity is often related to general biothreats and/or laboratory biosafety and biosecurity.

## 4. Discussion

### 4.1. Distribution of Records over Space and Time

While 54 countries were referenced in the literature review findings, the distribution of records across countries and regions showed disparities. One-third of records related to biosecurity initiatives were implemented in Oceania (specifically, Australia and New Zealand), while the African region was only represented by 9% of records. This observation was also applicable to the subgroups and domains identified. Biosecurity-related “tools”, for example, were often associated with Europe; this is most likely due to a large part of records referring to the Biocheck.UGent tool (*n* = 10/24 tools in Europe) [40,41]. Due to the dominance of records referring to Australia and New Zealand, we have decided to separate them from the original geoscheme region “Oceania”.

When categorising countries by income status, HICs were more commonly represented (71%). It is recognised that research is not addressing health-related needs in a balanced way and that HICs produce a disproportionately large number of publications compared to the proportion of the global population living in these countries and the disease burden affecting them [42]. Further, research highlights that global health efforts are also often biased towards HICs [43]. Our review findings support the notion that current research on biosecurity-related initiatives mainly relate to HICs and focus on their health concerns, potentially resulting in insufficient attention to the biosecurity concerns in low- or middle-income countries that are specific to the local context [44,45].

It is also recognised, however, that countries like Australia and New Zealand are more highly represented since they have a longstanding history of strong biosecurity systems (including quarantine, pre-border controls, and trade restrictions), with unique wild animal and plant species, a large agriculture sector and disease freedom statuses to protect, and a high interest in approaches such as One Health [46,47,48,49]. Conversely, Latin America was underrepresented, with only five records. We recognise that this is likely to be explained by the fact that English was the only language used for the initial search due to resource limitations, potentially resulting in language bias.

Overall, the amount of biosecurity-related literature published is steadily increasing. This has also been found in similar publications on biosecurity related to livestock [2,50]. While acknowledging the importance of research and publications to inform evidence-based policies, it is important to carefully consider potential biases. For instance, Southwell et al. found that past infectious disease outbreaks have conditioned and constrained public response to novel viral diseases [51]. This might explain temporal peaks and so called ‘roller coaster’ funding cycles that occur in response to each new emerging infectious disease threat [52]. Such funding cycles can potentially influence the sustainability of biosecurity efforts: A report suggested, for example, that fragmented and ad hoc public or external donor funding in response to health emergencies can create distortions in livestock systems that prevent the emergence of more sustainable alternatives for basic livestock health service delivery by the private sector [53,54].

The overall number of records identified in this review is rather small compared to other reviews, such as by Renault et al. [52]. This may be partly explained by the use of restrictive eligibility criteria. A large part of the records found during the initial search referred to specific biosecurity measures at the farm level, which were not part of this review’s focus and were, thus, excluded, unless they referred to a specific program, tool, or other interventions. Access to biosecurity-related information or evidence of initiatives might be biased due to not being available open access or not published and available online as a result of inadequate resources and/or conflicts of interest by donors, researchers, governments, private sectors, or other parties [55,56]. Similar limitations have been found for the reporting of disease events that may be jeopardised by competing interests, such as trade restrictions [57], which have an obvious implication for maintaining strong biosecurity in a globalised world.

### 4.2. Emphasis on Traditional Livestock Species and Hazards

The livestock species mentioned in the records showed a tendency towards the “traditional” livestock species (being large ruminants, pigs, and poultry). For large ruminants, no records were captured referring to non-cattle ruminants, such as yaks or camels. Further, only a minority of records referred to small ruminants, equines, and farmed or exotic animals for trade. This might indicate a need for more careful consideration since emerging infectious disease threats involving such species are of increasing importance [58,59]. In regard to exotic animals or wildlife more generally, it is worthwhile noting that the understanding of the terms “livestock” and “wild animals” is often different among people, which can make a more evidence-based discussion difficult. In the context of COVID-19 for example, most cases of “wild animals” traded in markets were not from free-living wildlife populations, but from farmed or captive populations that are not fully domesticated, but are kept in domestic conditions [60]. As such, they may better be classified as wildlife farming systems, which share similar characteristics with common livestock systems, including breeding, husbandry, and management practices [61,62,63]. The same reflection might apply to aquaculture, which is frequently separated from livestock in terms of the legal, financial, and broader institutional setup of the public sector [64,65]. Such a farming systems view on farmed livestock and captive wildlife may provide opportunities for biosecurity, as farming systems are the result of human design (as opposed to natural ecosystems that are outside human control) [66].

Our results further indicate limited consideration of biosecurity across the entire value chain, with less than 10% of included records referring to biosecurity implemented post-production. Maintaining biosecurity at farms (keeping disease agents from moving into and out of farms) is important, but there is also a need to pay attention to biosecurity (risk reduction) in supply, production, transport, and marketing chains [67]. This might suggest a neglection of disease transmission risks at those post-production stages, including the wide range of stakeholders involved, such as slaughterers, traders, retailers, or consumers. Similarly, most records related to viral diseases indicate a potential bias or disregard of other hazards, such as bacteria, fungi, prions, pests, and parasites, which also arguably pose considerable biosecurity risks [68,69]. Only a few records referred to foodborne bacterial diseases, tick-borne diseases, or AMR/AMU. The few records on “AMR/AMU” were from Europe (80%) and Northern America (20%), while this topic is arguably of equal, if not greater, importance in the Global South, since current estimates of deaths caused by or associated with AMR are expected to be highest in sub-Saharan Africa in contrast to HIC regions [70].

### 4.3. Enabling Environment for Biosecurity

The health threat of AMR illustrates the complex challenges related to biosecurity, particularly in LMICs. It is recognised that factors beyond the production level influence AMR, such as insufficient control and regulatory mechanisms for drugs, over-the-counter dispensing of antibiotics, limited resources and technical capacities, lack of awareness, and substandard or counterfeit drugs [71,72]. Those considerations indicate a wider enabling environment needed for effective biosecurity implementation, which has also been highlighted as a key challenge in disease surveillance, preparedness, and response [73]. While many efforts currently focus on improving disease surveillance and research, greater attention is needed to understand how to improve biosecurity infrastructure (e.g., roads, cold chain, diagnostics, clinics, etc.) [74]. This is a particular challenge that relates to the geographic disparity mentioned before between HICs and LMICs and emphasises an interconnection of biosecurity efforts across value chains within, but also across, countries globally. Several authors have, accordingly, highlighted that biosecurity and sustainable health systems are interconnected and require a more holistic approach [73,75,76,77] and true global partnership [78] that takes into consideration the specific social and economic contexts of the region or country [79].

Related to the wider enabling environment for biosecurity are also “prerequisites”, which form the foundation of control approaches to general hazards and are often known as good practices in production, hygiene, manufacturing, or sanitation standard operating procedures. In fact, prerequisites are frequently ignored or bypassed in favour of advanced hazard-specific programs, such as hazard analysis and critical control points (HACCP). FAO and World Health Organization (WHO) guidance states that without prerequisites, a risk-based system such as HACCP will fail [80]. A related problem of too advanced control approaches is also that they often involve an unaffordable start-up cost [81] or ignore the needs of stakeholders at the community and local levels, whose engagement is a crucial ingredient to ensure better assessment and management of animal, plant, and environment health [82]. The stakeholder engagement should also take into account the underlying business models, as research shows that private actors are more likely to comply with biosecurity if they see their economic viability [6,83]. Stepwise approaches are a practical way to build stakeholder engagement, but they require flexibility, which is another important element, as most biosecurity-related legislations or policies assume effective enforcement immediately [84] and often do not consider the possibility of non-compliance [85]. As such, they fail to address that many policies require significant amounts of time, funding, and human resources to be successfully implemented—especially for small-scale actors, who may not have the financial resources to comply with biosecurity standards.

### 4.4. Survey Results

While the number of responses retrieved from the online survey was limited, they have been included to triangulate, complement, and validate the literature review findings. Both the literature review and the survey have shown a strong focus on viral diseases and traditional livestock species. Similar to the findings of the content analysis from the review, essential elements for good biosecurity at the community level included knowledge and technical resources (including emergency management, guidelines, capacity, and practices), but also awareness and engagement (e.g., partnerships, cooperation, multistakeholder and participatory approaches). Engaging SMEs offers an added value to answering complex research questions by enhancing the validity and reliability of the findings.

### 4.5. Need for More Coherent Terminology

Through this review, different definitions and understanding of the term “biosecurity” became apparent, including laboratory biosafety and biosecurity, farm biosecurity, bioterrorism, infection prevention and control (IPC), and, more broadly, water, sanitation, and hygiene (WASH). In fact, the understanding of “biosecurity” has changed over time and has led to several definitions that exist in parallel today [2]. As for “livestock biosecurity”, a recent article discussed the current situation and challenges of defining and understanding the term in detail [50]. The authors found that the most popular biosecurity definition was the one that conceptualised the rules of 5B’s (bio-exclusion, bio-containment, bio-compartmentation, bio-prevention, and bio-preservation) and concluded with the need for an operational definition covering animal health, but also public health [50]. While the purpose of this review was not to analyse definitions, the need for more careful and consistent terminology is underlined, which could be supported by relevant international agencies, such as the Quadripartite consisting of the FAO, WHO, World Organization for Animal Health (WOAH), and the Environmental Programme (UNEP).

As the aim of this review was to identify possible strategic recommendations to support the development of the PMP-TAB, a broad definition of biosecurity was used, considering it as “[…] a strategic and integrated approach to analysing and managing relevant risks to human, animal and plant life and health and associated risks to the environment” [4]. While this definition was advantageous to identify a broad number of records, it also created confusion among survey participants. One respondent criticised overlaps of this definition with other holistic approaches, such as One Health, which highlights the difficult balance between conceptual, all-inclusive approaches and keeping them practical, understood, and operational at the local level [86].

### 4.6. Need for a Global Approach to Biosecurity

As mentioned above, this review did not identify any comprehensive frameworks to successfully scale up biosecurity, despite the increasing number of publications and global relevance of the topic. Therefore, a global and holistic approach, such as the previously mentioned PMP-TAB, is warranted and timely. This need has also been acknowledged recently by an article on the global governance of One Health and a related fragmented, global, multilateral health security architecture, which specifically called for “adopting a One Biosecurity approach” [87]. Similar initiatives have evolved in response to related global health challenges (e.g., the Global Health Security Agenda). Progressive or stepwise approaches exist at the global scale for specific diseases, health challenges, or sectors [88,89,90]. Potential elements to develop such a stepwise framework for strengthening or improving biosecurity already exist today, including Chapter 6.5 from the WOAH Terrestrial Animal Health Code on “Biosecurity procedures in poultry production” [91] or other existing entities and standards (e.g., International Organization for Standardization, Codex Alimentarius Commission, Sanitary and phytosanitary measures). Nevertheless, more research on integrating those into a global framework for scaling up biosecurity globally is needed.

### 4.7. FAO-PMP-TAB

As outlined in the published concept note of the PMP-TAB [10], the elements and principles guiding its development were included and based on other previous works, such as the FAO Biosecurity Toolkit [4], other progressive management pathways (including PMP-AMR and PMP for improving aquaculture biosecurity), and discussions with SMEs. The core component of the PMP-TAB of knowledge and evidence is also identified as a key strength in this review, specifically, as a focus on evidence-based approaches. In addition, elements such as “legislation” and “financing” or “capacity building” identified in this review are also reflected in the PMP-TAB concept note. However, other elements, like “participatory approaches” (identified as a strength) or “technologies” (frequently mentioned as opportunity), might be considered for inclusion in greater detail. While technology might be easily added to one core component, elements such as participatory approaches or private–public partnership require a more process-oriented thinking of how to integrate them best in a pathway. Some important elements from the SWOT analysis should be carefully considered, too, including, for example, the frequently mentioned weakness of the “standardization or generalization” of biosecurity-related guidance, which might call for more tailored, local approaches. Similarly, the threat of “trade” interests or “demographic change”, i.e., shifts in population composition that might affect biosecurity, need to be considered more carefully.

### 4.8. Strengths and Limitations of the Scoping Review

Regarding the overall methodology of content analysis, we are aware of the criticism by some scholars that consider the process of coding and counting frequencies of specific textual information as too simplistic and weak in terms of analytic value. However, this approach was appropriate, in our view, to assess the complex, current body of literature on biosecurity-related initiatives and their characteristics and information. Here, an inductive approach of content analysis was preferred over the alternative deductive approaches, which would start with preconceived codes or categories derived from prior relevant research [92,93]. Moreover, such codes or categories did not exist prior to this review, to our knowledge. As illustrated by the domain of “others” (Figure 5), a broad classification merging various topics was considered large in our analysis. This scoping review may offer a possibility for future research to capture and use different classification and subgroups and focus on more precise research questions in order to avoid broad and complex classifications.

While the current review used qualitative content analysis, which aims to “systematically describe the meaning” of materials the researcher specified from the research question [94], it does not focus on identifying relationships among categories or theory building. The key question here to investigate further might be the question of how biosecurity systems are developed and strengthened. Therefore, additional research may take advantage of grounded theory, which is appropriate when no theory exists or when a theory exists that is too abstract to be tested [95,96]. Further, while this review focused on the rather narrow technical field of biosecurity, it may be necessary to go into non-biosecurity fields, such as management science or governance science, to understand how to scale up systems sustainably.

Another limitation of this review is that it is based only on the screening of titles and abstracts (or similar texts). Given the various definitions and understanding of biosecurity, titles and abstracts may provide limited information on the understanding of biosecurity and the SWOTs. While uncertainties of the inclusion and coding of records and findings were discussed and validated by various members of the research team, a second reviewer should be considered for future complex reviews to limit potential subjective bias.

## 5. Conclusions

This review was conducted to identify, characterise, and analyse existing biosecurity initiatives at the local-to-global level. A comprehensive, global framework to scale up biosecurity was not identified through the review process, which highlights the need for the development of frameworks like the PMP-TAB. Considerable disparities in the geographic distribution of evidence related to biosecurity initiatives and differences in definitions for biosecurity were found, which call upon international organisations such as FAO to spearhead efforts towards a global framework to progressively support the improvement of biosecurity under a unifying One Health approach. Through content analysis of titles and abstracts and consultations with SMEs, different elements and SWOTs of current biosecurity systems, initiatives, programs, or projects were identified. While the generated information can inform the design of biosecurity systems, we acknowledge that there is no one-size-fits-all approach to biosecurity. Based on the findings of this review and similar efforts mentioned in the discussion, the following general recommendations have been developed. While they may not be exhaustive, they can be useful to consider when developing approaches or frameworks to progressively improve biosecurity.

- Ensure a clear definition of “biosecurity”, including scope and objectives. During the initial search and selection of terms for the literature review, several definitions and understandings of “biosecurity” were identified, which can hinder a harmonious understanding across sectors and disciplines.
- Include all relevant stakeholders along the value chains of biosecurity relevant fields, including public and private stakeholders, as well as formal and informal actors, in the design of biosecurity initiatives. The results show that multistakeholder approaches and public–private partnerships were among the most identified elements in the review and considered in the survey.
- Consider biosecurity at each level, from the local (e.g., enterprise) to the national and global (e.g., borders) levels. Different biosecurity-related topics appeared at different levels, implying that priorities vary and might, thus, deserve a more careful or weighted consideration in the development of pathways (e.g., community engagement and legislation appeared as important elements, with engagement appearing more frequently at the local level, while the legislation appeared in more records related to the national level).
- Start with the basics or prerequisites of biosecurity for general hazards as the foundation of disease control and prevention (e.g., good hygienic practices). While most records focused on few hazards, research highlights that prerequisites are often ignored or bypassed in favour of too advanced control approaches.
- Consider the wider enabling environment necessary for biosecurity. The results show that the institutional capacity, including, for example, the financial resources, legislation, and workforce, as well as health infrastructure (e.g., roads, cold chains, laboratories), is a critical element in the wider enabling environment for biosecurity.
- Offer tailored and flexible approaches, as biosecurity implementation should be specific to the local context and its respective environment and systems. This review identified generalised standards or “one-size-fits-all” solutions as key weaknesses of current biosecurity. The findings also highlighted that policies should be flexible and allow for delayed uptake instead of assuming perfect immediate enforcement.
- Rely on an all-inclusive approach to ensure that all hazards are sufficiently covered. The results have shown that most records referred to biosecurity initiatives being specific to a single disease and related to traditional livestock species, and failing to recognise that a wide range of hazards are important and can be prevented by applying biosecurity. Other hazards and aspects that are important, but rarely considered, include neglected diseases, farmed game, and informal value chains.
- Make the uptake of biosecurity simple and attractive for private actors. The feasibility, practicability, and economic incentives of biosecurity measures play an important role for stakeholders at the enterprise and local levels, as shown in the review, as well as in the survey results.
- Consider all relevant stages of value chains across different systems. The majority of records in this review referred to production stages only. Post-production reflects an equally important aspect to ensure strong biosecurity systems and should be consistently considered.
- Take a multidisciplinary approach when developing frameworks for progressive improvement of biosecurity. This review focused on existing initiatives only within the field of biosecurity related to health, but many records have highlighted that solutions may also be found in research related to other fields, such as in good governance, social, or management sciences.
- Enhance the sustainability of biosecurity initiatives by considering factors related to economic, social, and environmental sustainability. The findings suggest that biosecurity efforts might be compromised by fragmented and ad hoc public or external donor funding in response to health emergencies that can prevent the emergence of more sustainable alternatives for basic livestock health service delivery.

## Figures and Tables

**Table 1:** The classification of domains described with their content focus and with a reference example. Please see Table S4.

Domain	Focus	Reference Example
AMR/AMU	Antimicrobial resistance, antimicrobial usage	[31]
Aquaculture	Aquaculture, fisheries, marine subjects	[32]
Awareness and engagement	Focus on engagement, awareness raising, community practices	[33]
Disease control	Focus on (specific or non-specific) animal diseases, its control, or prevention measurements	[34]
Livestock	Related to farm animal husbandry, veterinary service related to livestock (including small ruminants, large ruminants, poultry, pigs, and bee farming [35])	[27]
Plant and environmental health	Plant, environment, forestry, particular plant pests and control	[36]
Public health	Focus on human public health services (incl. COVID-19 pandemic)	[37]
Trade/border	Trade, trade regulations, border controls	[38]
Other	Agriculture, food safety and security, biosafety and laboratory biosecurity, wildlife, biorisks, legislation and governance and policy, regulatory mechanisms, multilateral partnerships, tourism, technology/model/intelligence system, non-plant pests and control (incl. invasive species, sentinel plants), social science/behaviour science, bioenergy, climate and weather	[39]

## Data Availability

Data are contained within the article or Supplementary Material.

## Supplementary Materials

The following supporting information can be downloaded at: https://www.mdpi.com/article/10.3390/ani13162672/s1, Supplementary File S1. Detailed Information on the Methodology of the Scoping Review and the Survey Questionnaires (incl. Tables S1–S6). Supplementary File S2. List of All Included Records. References [17,35] is cited in the supplementary materials.
