# Supplementary material for: Characterising Biosecurity Initiatives Globally to Support the Development of a Progressive Management Pathway for Terrestrial Animals: A Scoping Review
Source: Animals (Basel). 2023 Aug 19;13(16):2672. doi: 10.3390/ani13162672 (PMC10451226; doi:10.3390/ani13162672)
Supplement: Supplementary file 1 [file animals-13-02672-s001.zip › animals-2509491-SupplementaryFileS1-S2_18.08.23.pdf]

Supplementary File S1 - Detailed Information on the Methodology of the Scoping Review and the Survey Questionnaires

1. *Detailed methodology/ protocol*

1.1. Scoping review

The review team consisted of one researcher (NM), who developed the protocol including the research questions, search strategy, eligibility criteria, data characteristics/classifications, screening, data extraction and summery in close consultation with the other authors (MM, AR, MH, SvD and MH).

1.1.1. Research questions

- What biosecurity relevant literature exists at global scale and how is it distributed over time and space?
- What biosecurity-related aspects or contents may be important for sustainable biosecurity systems?
- What are the strengths, weaknesses, opportunities and threats of existing biosecurity systems, initiatives, programs, or projects?
- How can the generated information support the countries in progressively improving biosecurity in production systems and value chains, through the application of evidence based, One Health approaches?

1.1.2. Databases used

- https://pubmed.ncbi.nlm.nih.gov/ (accessed on 18.04.2022)
- https://www.webofscience.com/wos/woscc/basic-search (accessed on 18.04.2022)
- https://www.fao.org/publications/search/en/ (accessed on 18.04.2022)
- https://scholar.google.de/ (accessed on 18.04.2022)
- https://www.ilri.org/publications (accessed on 18.04.2022)
- https://www.worldbank.org/en/research (accessed on 18.04.2022)

1.1.3. Keywords and search query

As stated below, the following keywords were used for performing the scoping literature review (Table S1), while the search query was performed on the 18 April 2022 and resulted in a total of 1350 literature reports (Table S2).

Table S1. Keywords used in this literature review.* refers to open end of the keywords

**Keywords 1 [title]**	**Keywords 2 [title and/or abstract]**	**Keywords 3 [title and/or abstract]**
Biosecurity	Standard*<br>Guideline*<br>Approach*<br>Practic*<br>Implement*<br>Measurement*<br>Pathway*<br>Tool*	Animal<br>Agricultur*<br>Livestock<br>Disease*<br>Zoono*<br>Aqua*<br>Fish*<br>Public Health

	Initiative*<br>Program*<br>Compliance<br>System*	AMR<br>Wildlife<br>Plant*<br>Forest*<br>Food safety

Table S2. Search query and resulting publication counts in this literature review.

**Database**	**Search query**	**Searches 18.04.2022**
PubMed	(biosecurity[title]) AND<br>((standard*[tiab]) OR (guideline*[tiab]) OR (approach*[tiab]) OR (practice*[tiab]) OR (implement*[tiab]) OR (measurement*[tiab]) OR (pathway* [tiab]) OR (tool*[tiab]) OR (initiative*[tiab]) OR (program* [tiab]) OR (compliance [tiab]) OR (system*[tiab])) AND<br>((animal[tiab]) OR (agriculture*[tiab]) OR (livestock[tiab]) OR (disease*[tiab]) OR (zoono*[tiab]) OR (aqua*[tiab]) OR (fish*[tiab]) OR (public health[tiab]) OR (AMR[tiab]) OR (wildlife[tiab]) OR (plant*[tiab]) OR (forest*[tiab]) OR (food safety[tiab]))	384
Web of Science	TI=(“biosecurity”) AND<br>(TS= (standard* OR guideline* OR approach* OR practice* OR implement* OR measurement* OR pathway* OR tool* OR initiative OR program* OR compliance OR system*)) AND<br>(TS=(animal OR agricultur* OR livestock OR disease* OR zoono* OR aqua* OR fish* OR “public health” OR amr OR wildlife OR plant* OR forest* OR “food safety”)	673
FAO	Biosecurity (title only)	56
Google scholar	allintitle: biosecurity system OR approach	210
ILRI	Biosecurity (keyword)	18
World Bank	Biosecurity (keyword, basic search)	09
**TOTAL**		**1350**

The following literature search was performed by one person (NM) in close consultation with other authors (MM, AR, SvD, MD, and MH) and consisted of the following steps:

1. Number of records identified through database search, other sources (no exclusion)
2. Number of records after duplicates removed in EndNote (Version 20)
3. Export of reference into Rayyan (Rayyan Systems Inc.) [1]
4. First screening of abstracts and title for eligibility criteria (Table S3)
    - Screening of title and abstracts
    - Labeling of publication within Rayyan software
    - Exclusion reasons: wrong population, wrong intervention, wrong outcome or others. Also, publications were excluded if no title or abstract (or similar text) was available.
5. Export publication list into an excel file (Microsoft Office Excel)
6. Second screening of abstracts and title for information extraction in an excel file (Table S4):
    - Descriptive information
    - SWOT analysis (if applicable Table S5)

Table S3. Exclusion and inclusion criteria used regarding the PICO checklist as proposed in the PRISMA guidelines.

**Area**	**Category**	**Inclusion criteria**	**Exclusion criteria**
Implementation of biosecurity standards and tools	Population	• Biosecurity measurements/ frameworks/ systems in relation to sanitary, phytosanitary and zoonsanitary measures applied in food and agriculture regulatory systems<br>• Global (LMIC and HIC)<br>• Including all stakeholders of livestock value chains (farmers, traders/transporters, slaughters, retailers, consumers, ministries, communities, industry)<br>• Including different animal-production systems (intensive, extensive, small-scale, nomadism& transhumance)<br>• Including endemic areas of specific diseases	• Biosecurity measurements in relation to laboratory work/ laboratory animals<br>• Biosecurity measurements in relation to biological weapons and/or bioterrorism
	Interventions	• Biosecurity systems, approaches, programmes, policies, strategies, regulations, legislations, laws, pilots<br>• Also referring to other prevention measures related to biosecurity practices such as on waste (water) management, vaccination<br>• Biosecurity implementation on all intervention levels (local, national, global)	• Evaluation of individual, single on-farm or off-farm level biosecurity practices (except if used for change strategies)<br>• Outbreak related disease surveillance associated with biosecurity measures<br>• Only risk/hazard/pest/disease identification or assessment or management<br>• Background information and/or only theoretical recommendations (if not in accordance to a particular program/ case)
	Outcome	• Effectiveness and/ or efficiency of implementation (Change of disease outbreaks& their costs,	• Only determination of cost efficiencies<br>• Only evaluation of stakeholder current

		change of stakeholder behavior) on system level<br>• Reports on SWOTs: strengths, weaknesses, opportunities, threats<br>• Reports on applicability, sustainability	behavior, knowledge, attitudes (except behavior change strategies/interventions) unless referring to biosecurity project
	Others	• English language<br>• Other languages if found by English keywords<br>• Grey literature included (including book chapters)<br>• Also secondary literature included if referring to biosecurity system/ initiative/ programme/ project	• Book reviews<br>• Whole books

Table S4. Documentation tracking of included literature.

**Data extraction/classification**	**Focus**
Title	
Year	
Authors	
Institution (only first authors‘ first named institution)	
Document (article, conference contribution, review, report, others)	Article= peer-reviewed research article;<br>Conference contribution= abstract, presentation;<br>Review= secondary literature (review article);<br>Report= reports;<br>Others= thesis, brief, book chapter, website article, letter to the editor, press article, factsheet.
Subgroup (Program, Tool, General, Strategy/ system/ legislation)	Program= program, programme, project, initiative;<br>Tool= tool, material, diagnostics, technology (laboratory or computational), artificial intelligence, model;<br>General= topics without specific focus on a program, country/region, tool or case; related to technology, trade/border, disease, surveillance, pest, agriculture, behavior science, practices;<br>Strategy/System= strategies, systems, approaches, frameworks, governance, policy, legislations.
Region	based if country/regional reference in the text appeared (no reference to authors affiliation); based on UN M49 geoscheme (https://unstats.un.org/unsd/methodology/m49/ accessed on 12.06.2023) with certain modifications: Africa, Asia, Europe, Australia/ New Zealand (incl. Tokelau), Oceana (incl. Melanesia, Micronesia, Polynesia), Latin America (incl. Caribbean, Central and South America), Northern America, Global and no region.

Region specified (country, not for analyses)	
Domain (AMR/AMU, aquaculture, awareness& engagement, disease control, livestock, plant and environmental health, public health, others)	AMR/AMU= antimicrobial resistance, antimicrobial usage;<br>Aquaculture= aquaculture, fisheries, marine subjects;<br>Awareness& engagement= focus on engagement, awareness raising, community practices;<br>Disease Control= focus on (specific or non-specific) animal diseases, its control or prevention measurements;<br>Livestock= related to farm animal husbandry, bees farming, veterinary service related to livestock (livestock as defined by FAO [2]);<br>Plant and environmental health= plant, environment, forestry, particular plant pests& control;<br>Public Health= focus on human public health services (incl. Covid19 pandemic);<br>Trade/ border= Trade, trade regulations, border controls;<br>Others= agriculture, food safety& security, biosafety& laboratory biosecurity, wildlife, biorisks, market/ trade/ border, regulatory mechanisms, policy/ legislation/ strategy/ direction, multilateral partnerships, tourism, technology/ model/ intelligence system, non-plant pests& control (incl. invasive species, sentinel plants), social science/ behavior science, bioenergy, climate& weather
Animal groups (large ruminants, small ruminants, pigs, poultry, others)	Others= horses, arthropods, wildlife animals
Disease information (not for analyses)	Name of pathogen focus
Disease focus (no disease focus, virus, bacteria, parasite)	
Level of action (local, national, regional/global, more than one level, no level)	Local= based on community/ district/ municipality/ state level;<br>National= based on country-level<br>Regional/global= based on regional of global focus
Mode of action (practical, theoretical, both)	Practical= practical testing of a tool or related to a particular activity/program/project/implementation/study/field application<br>Theoretical= theoretical report, related to an approach or system description and not related to a specific program/project/implementation, may have a general reference, may be related to only recommendations/outlook<br>Both= reference with practical and theoretical input (e.g. reviewing previous legislations under a particular strategy, case studies to evaluate practical approaches)
Initiative name (not for analyses)	
Strenght1	Table S5

Strenght2	
Strenght3	
Opportunity1	
Opportunity2	
Opportunity3	
Weakness1	
Weakness2	
Weakness3	
Threat1	
Threat2	
Threat3	

Table S5. Top 10 of each SWOT (strength, weakness, opportunity, threat) code used thought this review for the subgroups “Program”, “Strategy/System”, and “General”.

**SWOT**	**Code**	**Keywords**
**Strength**	Multistakeholder	partnerships between governments and industry; enabling participants; collaborative; relevant stakeholders; stakeholder; collaboration; partners
	Participatory process	co-created; participatory approach; buy-in; participation; participatory training
	Capacity building	build capacity; community science experts; training; capability and capacity; instructions; educational; capacity development; strengthening capacity; education
	Evidence based	science based; risk analysis; novel analytical tools; structured tool; Evidence
	Characteristics	access; availability; dynamic; flexible; simple; comprehensive; specific; standardized
	Time	timely; rapid; quickly; long-term; constant
	Knowledge& information sharing	information; knowledge; knowledge best practices
	Awareness& engagement	engagement; awareness
	Surveillance& reporting	practices; detection; surveillance
	Guidelines& SOPs	written biosecurity plan; standards; list to enhance practices; training material; protocols
**Weakness**	Compliance	acountability; willingness; compliance; only few participants; low adoption
	Awareness& perceptions	risk perceptions; values; awareness; perception
	Time	short-term; slow; sustainability
	Capacity building	training; capacity building; professional development
	Financing& funding	cost; funding; funds; economic
	Knowledge& information sharing	data management; information; knowledge
	Coordination, management	coordination; internal management; guidance
	Law& policy	legislation; policy; rights; law enforcement
	Regulatory mechanisms	regulatory instruments; regulatory mechanisms
	Evidence based	evidence-based; scientific evidence

	Standardization/ Generalization	unspecific; variety; insufficient inclusion
**Opportunity**	Upscaling	multiplier; model; the first step; which can adopt; scaling; upscaling
	Law& policy enforcement	policy; legislative; decision-making; ministries and agencies; governmental; government
	Surveillance& reporting	reporting system; surveillance; monitoring; detection
	Multistakeholder	different stakeholders; state and non-state actors; stakeholders; collaboration; commitment and support of a number of key industry and government stakeholders
	Financing& funding	cost-effectiveness; economic incentives; sustainable funding; financial; cost; funding
	Knowledge& information sharing	knowledge; information; knowledge partnerships; dialogue
	Time	sustainability; timeliness; quicker; long-term
	Technology	digital; technology; model; online
	Other trade-offs	reduced antimicrobial usage; animal welfare; food safety
**Threat**	Tourism, mobility	high human accessibility; tourism; travel; high mobility; movement*; transport
	Financing& funding	illegal; budget; resources; cost efficiency; financial
	Demographic change	urbanization; growing population; demographic context; demographics; landholders
	Government feedback	government feedback; government commitment; the constraints of operating in their official capacities<br>authorities
	Trade	trading; undeclared; incursion
	Disease outbreak	measels outbreak; outbreak
	Time	long-term; not quickly
	Compliance	is still not implementing sufficient; implementation of biosecurity measures
	Knowledge& information sharing	knowledge; information
	Coordination, management	governance; operational roles& responsibilities

Table S6. Examples of main questions in the biosecurity survey.

Q#	Part	Question	Response type
#Q1	Biosecurity initiatives/ programmes/ projects: Considering the broader biosecurity definition, please name biosecurity initiatives, programmes or projects you have experience with. Provide links/ resources if available. Feel free to refer to initiatives, programmes,	Biosecurity initiative/ programme/ project #1 – name	free text
#Q2		Biosecurity initiative/ programme/ project #1 - short description (objective, species, region, ...)	free text
#Q3		Biosecurity initiative/ programme/ project #2 – name	free text
#Q4		Biosecurity initiative/ programme/ project #2 - short description (objective, species, region, ...)	free text

#Q5	projects on different levels (e.g. producer level, market level, consumer level, national level, global level, ...) in a wider biosecurity definition (e.g. considering relevance to disease control, surveillance, food safety, public health, wildlife, veterinary services, policy& governance...)	Are you aware of any other biosecurity initiatives/ programmes/ projects?	yes/no
#Q6		If yes, please indicate the names and give a short description of the biosecurity initiative/ programme/ project if possible.	free text
#Q7	Biosecurity systems and approaches; Please consider the broader definition of biosecurity while answering following questions: In your view, …	…which are essential components of a good biosecurity system?	free text
#Q8		…what do you consider important for strengthening biosecurity on community/ producer level?	free text
#Q9		...what are opportunities to integrate the private sector in strengthening biosecurity systems?	free text
#Q10	SWOT analysis of a successful biosecurity initiative/ programme/ project: Choose one initiative/ programme/ project that has been successful from your point of view and complete the SWOT analysis.	Initiative/ programme/ project name:	free text
#Q11		Who is implementing the initiative/ programme/ project?	free text
#Q12		Who are the key stakeholders?	free text
#Q13		Implementation level:	Choose: Local, National, Regional/Global
#Q14		Domain:	Choose: Large ruminants, small ruminants, pigs, poultry, other (specify)
#Q15		Please highlight key successes or outcomes of the initiative/ programme/ project	free text
#Q16	SWOT analysis (Strengths, Weaknesses, Opportunities, Threats); For a general understanding of a SWOT analysis, please see FAO Biosecurity Toolkit (2007), p.115 (in PDF p.127)	Strengths (internal, positive)	free text
#Q17		Weaknesses (internal, negative)	free text
#Q18		Opportunities (external, positive)	free text
#Q19		Threats (external, negative)	free text
#Q20		What were critical points for its successful implementation?	free text
#Q21	SWOT analysis of a biosecurity initiative/ programme/ project that encountered challenges to meet it; Choose one initiative/ programme/ project that has not been successful from your point of view and complete the SWOT analysis.	Initiative/ programme/ project name:	free text
#Q22		Who is implementing the initiative/ programme/ project?	free text
#Q23		Who are the key stakeholders?	free text
#Q24		Implementation level:	Choose: Local, National, Regional/Global
#Q25		Domain:	Choose: Large ruminants, small ruminants, pigs,

			poultry, other (specify)
#Q26		Please highlight key successes or outcomes of the initiative/ programme/ project	free text
#Q27	SWOT analysis (Strengths, Weaknesses, Opportunities, Threats); For a general understanding of a SWOT analysis, please see FAO Biosecurity Toolkit (2007), p.115 (in PDF p.127)	Strengths (internal, positive)	free text
#Q28		Weaknesses (internal, negative)	free text
#Q29		Opportunities (external, positive)	free text
#Q30		Threats (external, negative)	free text
#Q31		What were critical points for its successful implementation?	free text
#Q32	Others	Please feel free to give us any other information resources and reference material that you deem useful for our work.	free text
#Q33		…your name…	free text
#Q34		…your email address.	free text

2. References

1. Ouzzani, M.; Hammady, H.; Fedorowicz, Z.; Elmagarmid, A. Rayyan — a web and mobile app for systematic reviews (https://www.rayyan.ai/). *Systematic Reviews* **2016**, *5*.
2. FAO. Livestock statistics: Concepts, definitions and classifications. Availabe online: https://www.fao.org/3/cb2461en/cb2461en.pdf (accessed on 18 June 2023).

Supplementary File S2 - List of All Included Records

#	Title	Year	Authors
1	A Coordinated, Risk-Based, National Forest Biosecurity Surveillance Program for Australian Forests	2022	Carnegie, A. J.; Tovar, F.; Collins, S.; Lawson, S. A.; Nahrung, H. F.;
2	Securing participation in global pork production networks: biosecurity, multispecies entanglements, and the politics of domestication practices	2022	Wang, C. M.
3	A bioavailable strontium (87Sr/86Sr) isoscape for Aotearoa New Zealand: Implications for food forensics and biosecurity	2022	Kramer, R. T. and Kinaston, R. L. and Holder, P. W. and Armstrong, K. F. and King, C. L. and Sipple, W. D. K. and Martin, A. P. and Pradel, G. and Turnbull, R. E. and Rogers, K. M. and Reid, M. and Barr, D. and Wijenayake, K. G. and Buckley, H. R. and Stirling, C. H. and Bataille, C. P.
4	Reconciling risk and responsibility on Indigenous country: bridging the boundaries to guide knowledge sharing for cross-cultural biosecurity risk management in northern Australia	2022	Kirsten Maclean, Cathy Robinson, Ellie Bock and Phil Rist
5	Using a DNA barcoding approach to facilitate biosecurity: Identifying invasive alien macrophytes traded within the South African aquarium and pond plant industry	2022	Niemann, HJ; Bezeng, BS; Orton, RD; Kabongo, RM; Pilusa, M; van der Bank, M;
6	Africa Sustainable Livestock 2050. Co-creating solutions for biosecurity and broiler business. Co-creating solutions for biosecurity and broiler business	2021	FAO
7	Improving Biosecurity: A Science-Based Approach to Manage Fish Disease Risks and Increase the Socioeconomic Contribution of the Nigerian Catfish and Tilapia Industries	2021	Khor, L; Delamare-Deboutteville, J; Chadag, V;
8	Changes in farmer animal health and biosecurity knowledge, attitudes and practices: Insights from Cambodia and Laos	2021	MacPhillamy, I.; Olmo, L.; Young, J.; Nampanya, S.; Suon, S.; Khounsy, S.; Windsor, P.; Toribio, J. A.; Bush, R.;
9	Animal welfare and biosecurity assessment: a comparison between Italian and Irish beef cattle rearing systems	2021	Fusi, F.; Lorenzi, V.; Franceschini, G.; Compiani, R.; Harper, V.; Ginestreti, J.; Ferrara, G.; Rossi, C. A. S.; Bertocchi, L.;
10	Interpretable machine learning applied to on-farm biosecurity and porcine reproductive and respiratory syndrome virus	2021	Sykes, A. L.; Silva, G. S.; Holtkamp, D. J.; Mauch, B. W.; Osemeke, O.; Linhares, D. C. L.; Machado, G.;
11	On-site hygiene and biosecurity assessment: A new tool to assess live bird stalls in wet markets	2021	Soon, J. M.; Wahab, I. R. A.;
12	4-H youth advance biosecurity at home and in their communities	2021	Smith, M. H.; Smith, W. A.; Meehan, C. L.;

13	Field application of biosecurity measures at small scale farmer level	2021	Lee, Hu Suk;
14	Advancing One Biosecurity to Address the Pandemic Risks of Biological Invasions	2021	Hulme, P. E.;
15	Mamalu Poepoe: Enhancing Hawaii's Biosecurity Through Interagency Collaboration	2021	Kaufman, L. V.
16	The Boundary of the Market for Biosecurity Risk	2021	Stoneham, G.; Hester, S. M.; Li, J. S. H.; Zhou, R.; Chaudhry, A.;
17	The Tasmanian biosecurity system-a strategically planned approach to delivering biosecurity outcomes	2021	Bishop, AC;
18	Assembling the team of 5 million: Socio-technical relations in Aotearoa-New Zealand's biosecurity system	2021	Edwards, Sarah
19	Tourism biosecurity risk management and planning: an international comparative analysis and implications for Ireland	2021	Melly, D.; Hanrahan, J.;
20	ddRAD sequencing: an emerging technology added to the biosecurity toolbox for tracing the origin of brown marmorated stink bug, Halyomorpha halys (Hemiptera: Pentatomidae)	2021	Yan, J. and VÃ©tek, G. and Pal, C. and Zhang, J. and Gmati, R. and Fan, Q. H. and Gunawardana, D. N. and Burne, A. and Anderson, D. and Balan, R. K. and George, S. and Farkas, P. and Li, D.
21	An Integrated Biosecurity Risk Assessment Model (IBRAM) For Evaluating the Risk of Import Pathways for the Establishment of Invasive Species	2021	Jamieson, L. E. and Woodberry, O. and Mascaro, S. and Meurisse, N. and Jaksons, R. and Brown, S. D. J. and Ormsby, M.
22	Are the EU biosecurity legislative frameworks sufficiently effective to prevent biological invasions in the Natura 2000 network?- A case study in Mediterranean Europe	2021	Baquero, R. A.; Ayllon, D.; Nicola, G. G.;
23	Elevating and Recognising Knowledge of Indigenous Peoples to Improve Forest Biosecurity	2021	Kuru, R.; Marsh, A.; Ganley, B.;
24	Forest Biosecurity in Canada - An Integrated Multi-Agency Approach	2021	Allison, J. D.; Marcotte, M.; Noseworthy, M.; Ramsfield, T.;
25	Policing biosecurity: police enforcement of special measures in New South Wales and Victoria during the COVID-19 pandemic	2021	Boon-Kuo, L. and Brodie, A. and Keene-McCann, J. and Sentas, V. and Weber, L.
26	Enacting and resisting biosecurity citizenship: More-than-human geographies of enrolment in a disease eradication scheme in Scotland	2021	Shortall, O.; Brown, K.;
27	U.S. Cattle Producer Adoption of Secure Beef Supply Plan Enhanced Biosecurity Practices and Foot-and-Mouth Disease Preparedness	2021	Pudenz, C. C.; Mitchell, J. L.; Schulz, L. L.; Tonsor, G. T.;
28	China's evolving biosafety/biosecurity legislations	2021	Cao, C.;
29	Forest biosecurity laws in South-East Asia: a review	2021	Healey, M. A. and Lawson, S. A. and Somany, S. and Tasen, W. and Ngoc, Q. D.

30	Plant-parasitic nematodes on turfgrass in Queensland, Australia, and biosecurity issues associated with the interstate transfer and eradication of southern sting nematode (Ibipora lolii)	2021	Stirling, G. R.; Stirling, A. M.; Eden, L.;
31	Deep Learning and Phenology Enhance Large-Scale Tree Species Classification in Aerial Imagery during a Biosecurity Response	2021	Pearse, G. D.; Watt, M. S.; Soewarto, J.; Tan, A. Y. S.;
32	Biosecurity at Cattle Farms: Strengths, Weaknesses, Opportunities and Threats	2021	Renault, V.; Humblet, M. F.; Pham, P. N.; Saegerman, C.;
33	Strengthening Biosecurity Preparedness through Enhanced Rapid Detection of African Swine Fever in Papua New Guinea - TCP/PNG/3706	2021	FAO
34	Opportunities for Transdisciplinary Science to Mitigate Biosecurity Risks From the Intersectionality of Illegal Wildlife Trade With Emerging Zoonotic Pathogens	2021	Aguirre, A. A.; Gore, M. L.; Kammer-Kerwick, M.; Curtin, K. M.; Heyns, A.; Preiser, W.; Shelley, L. I.;
35	Using Co-Design to Create Community Advocacy for Biosecurity Behavior Change	2021	Sherring, P.
36	Assessment of the Biosafety and Biosecurity Landscape in the Philippines and the Development of the National Biorisk Management Framework	2021	Destura, R. V.; Lam, H. Y.; Navarro, R. C.; Lopez, J. C. F.; Sales, R. K. P.; Gomez, Mifa; dela Tonga, A.; Ulanday, G. E.;
37	Forest Biosecurity Systems and Processes: An Indian Perspective	2021	Gupta, K.; Sankaran, K. V.;
38	Biosecurity practices and border control to stop the spread of African swine fever	2020	FAO
39	Antimicrobial usage, animal welfare and biosecurity in 16 dairy farms in Lombardy	2020	Ginestreti, J. and Lorenzi, V. and Fusi, F. and Ferrara, G. and Scali, F. and Alborali, G. L. and Bolzoni, L. and Bertocchi, L.
40	Using Biosecurity Measures to Combat Respiratory Disease in Cattle: The Norwegian Control Program for Bovine Respiratory Syncytial Virus and Bovine Coronavirus	2020	Stokstad, M.; Klem, T. B.; Myrmel, M.; Oma, V. S.; Toftaker, I.; Østerås, O.; Nødtvedt, A.;
41	A risk-based scoring system to quantify biosecurity in cattle production	2020	Damiaans, B.; Renault, V.; Sarrazin, S.; Berge, A. C.; Pardon, B.; Saegerman, C.; Dewulf, J.;
42	Biosecurity measures in European beekeeping	2020	Pietropaoli, M. and Ribarits, A. and Moosbeckhofer, R. and Koglberger, H. and Alber, O. and Gregorc, A. and Skerl, M. I. S. and Presern, J. and Bubnic, J. and Muz, M. N. and Higes, M. and Tiozzo, B. and Jannoni-Sebastianini, F. and Lubroth, J. and Cazier, J. and Raizman, E. and Zilli, R. and Bagni, M. and Della Marta, U. and Formato, G.
43	Herd typologies based on multivariate analysis of biosecurity, productivity,	2020	Kruse, A. B. and Nielsen, L. R. and Alban, L.

	antimicrobial and vaccine use data from Danish sow herds		
44	Development of a biosecurity assessment tool and the assessment of biosecurity levels by this tool on Japanese commercial swine farms	2020	Sasaki, Y. and Furutani, A. and Furuichi, T. and Hayakawa, Y. and Ishizeki, S. and Kano, R. and Koike, F. and Miyashita, M. and Mizukami, Y. and Watanabe, Y. and Otake, S. and P, J. E. T.
45	Participatory training and mobile phone assisted approaches for strengthening capacity of Uganda smallholder pigs value chains actors in biosecurity and disease control	2020	Dione, Michel M and Kangethe, Edwin and Dror, Iddo and Ndiwa, Nicholas N and Poole, Elizabeth J and Wieland, Barbara
46	Biosecurity levels of pig fattening farms from four EU countries and links with the farm characteristics	2020	Chantziaras, I. and Dewulf, J. and Van Limbergen, T. and Stadejek, T. and Niemi, J. and Kyriazakis, I. and Maes, D.
47	Impact of participatory training of smallholder pig farmers on knowledge, attitudes and practices regarding biosecurity for the control of African swine fever in Uganda	2020	Dione, M. M. and Dohoo, I. and Ndiwa, N. and Poole, J. and Ouma, E. and Amia, W. C. and Wieland, B.
48	A self-assessment tool to improve poultry farm biosecurity regarding avian influenza	2020	Grabkowsky, B. and Conraths, F. J. and Globig, A. and Wilke, A. and Denzin, N.
49	Detect-alert-deter system for enhanced biosecurity and risk assessment	2020	Atzeni, Michael and Muehlebach, John and Fielder, Darren and Mayer, David G
50	Biosecurity assessment of layer farms in Central Luzon, Philippines	2020	Tanquilut, N. C.; Espaldon, M. V. O.; Eslava, D. F.; Ancog, R. C.; Medina, C. D. R.; Paraso, M. G. V.; Domingo, R. D.;
51	Quantitative assessment of biosecurity in broiler farms using Biocheck.UGent in Central Luzon, Philippines	2020	Tanquilut, N. C. and Espaldon, M. V. O. and Eslava, D. F. and Ancog, R. C. and Medina, C. D. R. and Paraso, M. G. V. and Domingo, R. D. and Dewulf, J.
52	A united front against marine invaders: Developing a cost-effective marine biosecurity surveillance partnership between government and industry	2020	McDonald, J. I. and Wellington, C. M. and Coupland, G. T. and Pedersen, D. and Kitchen, B. and Bridgwood, S. D. and Hewitt, M. and Duggan, R. and Abdo, D. A.
53	Draft national strategy on aquatic animal health and biosecurity for the Federated States of Micronesia (2021- 2024)	2020	FAO, FSM, SPC
54	National Aquatic Animal Health and Biosecurity Strategy – FAO project TCP/MIC/3603/C2 for the federated states of micronesia	2020	MacKinnon, B. et al
55	Regulating Vessel Biofouling to Support New Zealand's Marine Biosecurity System - A Blue Print for Evidence-Based Decision Making	2020	Georgiades, E. and Kluza, D. and Bates, T. and Lubarsky, K. and Brunton, J. and Growcott, A. and Smith, T. and McDonald, S. and Gould, B. and Parker, N. and Bell, A.
56	How indigenous researchers are shaking up New Zealand’s biosecurity system via the	2020	Shadbolt, Melanie; Waipara, N; Black, Amanda;

	establishment of a Māori Biosecurity Network		
57	Recreational angling as a pathway for invasive non-native species spread: awareness of biosecurity and the risk of long distance movement into Great Britain	2020	Smith, E. R. C.; Bennion, H.; Sayer, C. D.; Aldridge, D. C.; Owen, M.;
58	Understanding general surveillance for biosecurity as a system	2020	Kruger, Heleen; El Hassan, Marwan; Stenekes, Nyree; Kancans, Robert;
59	The Epidemiological Framework for Biological Invasions (EFBI): an interdisciplinary foundation for the assessment of biosecurity threats	2020	Hulme, P. E. and Baker, R. and Freckleton, R. and Hails, R. S. and Hartley, M. and Harwood, J. and Marion, G. and Smith, G. C. and Williamson, M.
60	Program Chair Poster Pick: Healthy Farms Healthy Agriculture: A Web Hub for Biosecurity to Protect the Herd and Flock	2020	Smith, J. M. and Cummings, J. C.
61	Aquaculture biosecurity: Practical approach to prevent, control, and eradicate diseases	2020	Scarfe, A David and PaliÄ‡, DuÅ¡an
62	A decade of RCPAQAP Biosecurity improving testing for biological threats in Australia	2020	Lau, K. A. and Theis, T. and Kaufer, A. M. and Gray, J. L. and Rawlinson, W. D.
63	Evaluating the health of Australia's biosecurity system	2020	Schneider, Karen; Arndt, Edith; Baumgartner, John; Camac, James; Dodd, Aaron; Fraser, Hannah; Gomboso, Jay; Gibert, Anaïs; Kompas, Tom; Lane, Stephen;
64	Biosecurity situation assessment for livestock, plant and food areas in Bhutan. Online meeting report	2020	FAO
65	Our Australian biosecurity system needs a rethink for decade's challenges	2020	Still, C.
66	A Risk Analysis Framework for Prioritizing and Managing Biosecurity Threats	2020	Montibeller, G. and Franco, L. A. and Carreras, A.
67	Use of meteorological data in biosecurity	2020	Hemming, D. and Macneill, K.
68	Faba Bean Gall (Olpidium viciae K.) as a Priority Biosecurity Threat for Producing Faba Bean in Ethiopia: Current Status and Future Perspectives	2020	Meresa, B. K.; Gebremedhin, H. M.;
69	Supporting Urban Weed Biosecurity Programs with Remote Sensing	2020	Sheffield, K. and Dugdale, T.
70	Implementation of the GB Plant Health and Biosecurity Strategy 2014-2019 with foresight on a new strategy for 2020	2020	Spence, N.
71	The emergence of prioritisation systems to inform plant health biosecurity policy decisions	2020	MacLeod, A. and Lloyd, S.
72	Blurred lines: integrating emerging technologies to advance plant biosecurity	2020	Hu, Y. H. and Wilson, S. and Schwessinger, B. and Rathjen, J. P.
73	Missed Opportunities? Covid-19, Biosecurity and One Health in the United Kingdom	2020	Enticott, G. and Maye, D.

74	Biosecurity in multifunctional landscapes: challenges for approaches based on the concept of 'shared responsibility'	2020	Sinclair, K.; Curtis, A.; Freeman, P.;
75	Analysis of biosecurity-related policies governing the seaweed industry of the Philippines	2020	Mateo, J. P. and Campbell, I. and Cottier-Cook, E. J. and Luhan, M. R. J. and Ferriols, Vmen and Hurtado, A. Q.
76	Getting on board with biosecurity: Evaluating the effectiveness of marine invasive alien species biosecurity policy for England and Wales	2020	Shannon, C. and Stebbing, P. D. and Dunn, A. M. and Quinn, C. H.
77	Strengthening Capacity in Home Gardening, Healthy Food Awareness and Effective Biosecurity for Tokelau - TCP/TOK/3601	2020	FAO
78	Reasons for and barriers to biosafety and biosecurity training in health-related organizations in Africa, Middle East and Central Asia: findings from GIBACHT training needs assessments 2018-2019	2020	Rutebemberwa, E. and Aku, F. Y. and Al Zein, E. I. K. and Bellali, H.
79	An analysis of the current status and future of biosecurity frameworks for the Indonesian seaweed industry	2020	Kambey, C. S. B.; Campbell, I.; Sondak, C. F. A.; Nor, A. R. M.; Lim, P. E.; Cottier-Cook, E. J.;
80	Public private collaborations amidst an emergency plant disease outbreak: The Australian experience with biosecurity for Panama disease	2020	de la Cruz, J.
81	Emergency preparedness and contingency plans to aquatic animal disease emergencies	2019	Bondad-Reantaso, Melba G
82	Southeast Asia Strategic Multilateral Dialogue on Biosecurity	2019	Cicero, A. and Meyer, D. and Shearer, M. P. and AbuBakar, S. and Bernard, K. and Carus, W. S. and Chong, C. K. and Fischer, J. and Hynes, N. and Inglesby, T. and Kwa, C. G. and Makalinao, I. and Pangestu, T. and Sitompul, R. and Soebandrio, A. and Sudarmono, P. and Tjen, D. and Wibulpolprasert, S. and Yunus, Z.
83	Accelerating Action in Global Health Security: Global Biosecurity Dialogue as a Model for Advancing the Global Health Security Agenda	2019	Brizee, S. and Budeski, K. and James, W. and Nalabandian, M. and Bleijs, D. A. and Becker, S. J. and Wallace-Sankarsingh, S. and Ahumibe, A. and Agogo, E. and Ihekweazu, C. and Nikkari, S. and Ellis, M. and Gozzer, E. and Semesi, I. S. and Masuku, Z. M. and Ikram, A. and Tahir, F. and Makalinao, I. and Severance, H. A. and van Passel, M. W. J. and Cameron, E. E.
84	Using the Biocheck.UGent (TM) scoring tool in Irish farrow-to-finish pig farms: assessing biosecurity and its relation to productive performance	2019	da Costa, M. R. and Gasa, J. and Diaz, J. A. C. and Postma, M. and Dewulf, J. and McCutcheon, G. and Manzanilla, E. G.

85	Additive Bayesian Network analysis of associations between antimicrobial it consumption, biosecurity, vaccination and productivity in Danish sow herds	2019	Lopes, R. and Kruse, A. B. and Nielsen, L. R. and Nunes, T. P. and Alban, L.
86	Unmanned Aerial Vehicles and Biosecurity: Enabling Participatory-Design to Help Address Social Licence to Operate Issues	2019	Ogilvie, S. and McCarthy, A. and Allen, W. and Grant, A. and Mark-Shadbolt, M. and Pawson, S. and Richardson, B. and Strand, T. and Langer, E. R. and Marzano, M.
87	Adoption of Secure Pork Supply Plan Biosecurity by U.S. Swine Producers	2019	Pudenz, C. C. and Schulz, L. L. and Tonsor, G. T.
88	Assessment of Biosecurity Level in Pig and Poultry Production System in Vietnam Using Bio-check Technology Assessment of Biosecurity Level in Pig and Poultry Production System in Vietnam Using Bio-check Technology	2019	Cuc, Ngo Thi Kim; Tuan, Ha Minh; Dewulf, Jeroen; Postma, Merel; Dinh, Nguyen Cong;
89	Assessment of biosecurity practices and development of a scoring system in swine farms using item response theory	2019	Silva, G. S. and Leotti, V. B. and Castro, S. M. J. and Medeiros, A. A. R. and Silva, Apsp and Linhares, D. C. L. and Corbellini, L. G.
90	Biosecurity system reforms and the development of a risk-based surveillance and pathway analysis system for ornamental fish imported into Australia	2019	Hood, Y. and Sadler, J. and Poldy, J. and Starkey, C. S. and Robinson, A. P.
91	Building a sustainable aquaculture industry in South Africa: the role of biosecurity	2019	Christison, K. W.
92	Research on Current Situation and Path of Aquatic Biosecurity System Construction in Shandong Province	2019	Xu, Tao and Chen, Dongqing
93	Using behaviour science to maintain resilience of Queenslandâ€™s biosecurity system	2019	Keir, Stephanie
94	Biosafety and Biosecurity Challenges Facing Veterinary Diagnostic Laboratories in Lower-Middle Income Countries in Southeast Asia: A Case Study of Thailand	2019	Siengsanan-Lamont, J. and Kamolsiripichaiporn, S. and Ruanchaimun, S. and Patchimasiri, T. and Jongrakwattana, B. and Blacksell, S. D.
95	Connecting island communities on a global scale: case studies in island biosecurity	2019	Matos, J. and Little, A. and Broome, K. and Kennedy, E. and Sanchez, F. A. M. and Latofski-Robles, M. and Irvine, R. and Gill, C. and Espinoza, A. and Howald, G. and Olthof, K. and Ball, M. and Boser, C. L.
96	Managing the grand challenge of biological threats to food production: The importance of institutional logics for managing Australian biosecurity	2019	Bryant, M. and Higgins, V.
97	Reforming Biosecurity Legislation in Developing Countries: Increasing Market	2019	Black, R.

	Access or Maintaining Unequal Terms of Trade?		
98	A Collaboratory for the Distributed Collaborations Within a Biosecurity Laboratory and Across Different Organizations	2019	Li, J. and Zic, J.
99	The value of sentinel plants for risk assessment and surveillance to support biosecurity	2019	Mansfield, S. and McNeill, M. R. and Aalders, L. T. and Bell, N. L. and Kean, J. M. and Barratt, B. I. P. and Boyd-Wilson, K. and Teulon, D. A. J.
100	Improved biosecurity surveillance of non-native forest insects: a review of current methods	2019	Poland, T. M. and Rassati, D.
101	Optimal allocation of limited resources to biosecurity surveillance using a portfolio theory methodology	2019	Barnes, B. and Giannini, F. and Arthur, A. and Walker, J.
102	Enhancing plant biosecurity with citizen science monitoring: comparing methodologies using reports of acute oak decline	2019	Baker, E. and Jeger, M. J. and Mumford, J. D. and Brown, N.
103	Post-Border Forest Biosecurity in Australia: Response to Recent Exotic Detections, Current Surveillance and Ongoing Needs	2019	Carnegie, A. J. and Nahrung, H. F.
104	The post-politics of plant biosecurity: The British Government's response to ash dieback in 2012	2019	Tsouvalis, J.
105	Biosecurity institutions and the choice of contracts in international fruit supply chains	2019	Pavez, I. and Codron, J. M. and Lubello, P. and Florencio, M. C.
106	Emerging Stakeholder Relations in Participatory ICT Design: Renegotiating the Boundaries of Sociotechnical Innovation in Forest Biosecurity Surveillance	2019	Grant, A. and Pawson, S. M. and Marzano, M.
107	Participatory Impact Assessment following training of smallholder pig farmers on biosecurity for the control of African swine fever in Uganda	2018	Dione, Michel M
108	"Smart regulation" and community cooperation in Australia's modern biosecurity context	2018	Kruger, H.
109	Creating a framework for the prioritization of biosecurity risks to the New Zealand dairy industry	2018	Muellner, P. and Hodges, D. and Ahlstrom, C. and Newman, M. and Davidson, R. and Pfeiffer, D. and Marshall, J. and Morley, C.
110	Surveillance Data Highlights Feed Form, Biosecurity, and Disease Control as Significant Factors Associated with Salmonella Infection on Farrow-to-Finish Pig Farms	2018	Argüello, H. and Manzanilla, E. G. and Lynch, H. and Walia, K. and Leonard, F. C. and Egan, J. and Duffy, G. and Gardiner, G. E. and Lawlor, P. G.
111	Development of a risk assessment tool for improving biosecurity on pig farms	2018	Allepuz, A. and MartÃn-Valls, G. E. and Casal, J. and Mateu, E.

112	Contract bonus systems to encourage biosecurity adoption on small-scale broiler farms in Indonesia	2018	Komaladara, Aasp and Patrick, I. and Hoang, N.
113	Review of transmission routes of 24 infectious diseases preventable by biosecurity measures and comparison of the implementation of these measures in pig herds in six European countries	2018	Filippitzi, M. E. and Kruse, A. B. and Postma, M. and Sarrazin, S. and Maes, D. and Alban, L. and Nielsen, L. R. and Dewulf, J.
114	B-eSecure: electronic system to measure and improve biosecurity on pig farms	2018	Geurts, V and DÃaz, I and Collel-Surinach, M
115	Linking supply chain governance and biosecurity in the context of HPAI control in Western Java: a value chain perspective	2018	Indrawan, Dikky and Rich, Karl M and Van Horne, Peter and Daryanto, Arief and Hogeveen, Henk
116	Scoring biosecurity in European conventional broiler production	2018	Van Limbergen, T. and Dewulf, J. and Klinkenberg, M. and Ducatelle, R. and Gelaude, P. and Mendez, J. and Heinola, K. and Papasolomontos, S. and Szeleszczuk, P. and Maes, D. and Consortium, Prohlth
117	Strengthening biosecurity capacity of Palau FAO project tcp/pl/3601/c1	2018	Arthur, JR and Miles, J and Remengesau, I and Aguilar, Glenn and Ambatang, R and Sengebau, F and Isamu, T and Bondad-Reantaso, M and FAO
118	Development of a Regional Aquatic Biosecurity Strategy for the Southern African Development Community (SADC)	2018	FAO
119	Knowledge to action on aquatic invasive species: Island biosecurity - the New Zealand and South Pacific story	2018	Champion, P. D.
120	Extreme deep learning in biosecurity: the case of machine hearing for marine species identification	2018	Demertzis, K. and Iliadis, L. S. and Anezakis, V. D.
121	Sounding out pests: the potential of hydroacoustics as a surveillance and compliance tool in aquatic biosecurity	2018	Abdo, D. A. and Duggan, R. L. and McDonald, J. I.
122	Synthetic antibody: Prospects in aquaculture biosecurity	2018	Chong, C. M. and Low, C. F.
123	Key actors in driving behavioural change in relation to on-farm biosecurity; a Northern Ireland perspective	2018	Lahuerta-Marin, A. and Brennan, M. L. and Finney, G. and O'Hagan, M. J. H. and Jack, C.
124	Analysis of Australia's New Biosecurity Legislation	2018	Durant, S. and Faunce, T.
125	A Systems Approach to Agricultural Biosecurity	2018	Anand, M.
126	SurF: an innovative framework in biosecurity and animal health surveillance evaluation	2018	Muellner, P. and Watts, J. and Bingham, P. and Bullians, M. and Gould, B. and Pande, A. and Riding, T. and Stevens, P. and Vink, D. and Staerk, K. D. C.

127	CEBRA Project 1606E: Scoping the value and performance of interventions across the NZ Biosecurity system	2018	Robinson, Andrew; Brockerhoff, Eckehard; Ormsby, Michael;
128	A qualitative binary risk assessment model for regulating the biosecurity and environmental risk of endophytes	2018	Bromfield, K. E. and Corin, S. and Atapattu, A.
129	A Tool for Assessment of Animal Health Laboratory Safety and Biosecurity: The Safety Module of the Food and Agriculture Organization's Laboratory Mapping Tool	2018	Mouillé, B. and Dauphin, G. and Wiersma, L. and Blacksell, S. D. and Claes, F. and Kalpravidh, W. and Kabore, Y. and Hietala, S.
130	Benchmarking forest health surveillance and biosecurity activities for managing Australia's exotic forest pest and pathogen risks	2018	Carnegie, A. J. and Lawson, S. and Wardlaw, T. and Cameron, N. and Venn, T.
131	Biosecurity tool-set: optimising quarantine zones for eradication through consideration of Australia's success with plant pathogen eradications in horticulture	2018	Villalta, O. N. and Mebalds, M. and Edwards, J.
132	Opportunities and limitations for DNA metabarcoding in Australasian plant-pathogen biosecurity	2018	Bulman, S. R. and McDougal, R. L. and Hill, K. and Lear, G.
133	Biosecurity matters—challenges to New Zealand's biosecurity system©	2017	Hurr, K
134	Using scenario-based influence mapping to examine farmers' biosecurity behaviour	2017	Maye, D. and Enticott, G. and Naylor, R.
135	An internet-based bioinformatics toolkit for plant biosecurity diagnosis and surveillance of viruses and viroids	2017	Barrero, R. A. and Napier, K. R. and Cunnington, J. and Liefting, L. and Keenan, S. and Frampton, R. A. and Szabo, T. and Bulman, S. and Hunter, A. and Ward, L. and Whattam, M. and Bellgard, M. I.
136	Bulk tank milk antibody ELISA as a biosecurity tool for detecting dairy herds with past exposure to Mycoplasma bovis	2017	Parker, A. M. and House, J. K. and Hazelton, M. S. and Bosward, K. L. and Morton, J. M. and Sheehy, R. A.
137	Impact of participatory training on biosecurity protocols on the knowledge, attitudes and practices of smallholder pig farmers in Uganda	2017	Dione, Michel and Amia, Winfred and Akol, Joyce and Kungu, Joseph and Lule, Peter and Mayega, Lawrence and Nyapendi, Robinah and Kakinda, Mary Jo and Ouma, Emily
138	Enhancing biosecurity along Ugandas pig value chains to control and prevent African swine fever	2017	Dione, Michel M and Nantima, Noelina and Mayega, Lawrence and Amia, Winfred C and Wieland, Barbara and Ouma, Emily A
139	Compliance/non-compliance with biosecurity rules specified in the Danish Quality Assurance system (KIK) and Campylobacter-positive broiler flocks 2012 and 2013	2017	Sandberg, M and Dahl, J and Lindegaard, LL and Pedersen, JR
140	Quantification of biosecurity status in commercial poultry farms using a scoring system	2017	Chowdhury, EH and Das, PM and Islam, MR and Yamage, M

141	Engaging with risk (or not): shared responsibility for biosecurity surveillance and the role of community gardens	2017	Curnock, M. and Farbotko, C. and Collins, K. and Robinson, C. J. and Maclean, K.
142	Microorganisms: Good or Evil, MIRRI Provides Biosecurity Awareness	2017	Smith, D. and Martin, D. and Novossiolova, T.
143	Priorities for Australias biosecurity system: An independent review of the capacity of the national biosecurity system and its underpinning Intergovernmental Agreement	2017	Craik, Wendy and Palmer, David and Sheldrake, Richard
144	Year 1 Report: Valuing Australia's Biosecurity System, CEBRA Project 1607A-Milestone 6	2017	Dodd, A and Spring, D and Schneider, K and Hafi, A and Fraser, H and Kompas, T
145	Balancing collaboration with coordination: Contesting eradication in the Australian plant pest and disease biosecurity system	2017	McAllister, R. R. J. and Robinson, C. J. and Brown, A. and Maclean, K. and Perry, S. and Liu, S.
146	Australian plant biosecurity surveillance systems	2017	Anderson, C. and Low-Choy, S. and Whittle, P. and Taylor, S. and Gambley, C. and Smith, L. and Gillespie, P. and Locker, H. and Davis, R. and Dominiak, B.
147	Knowledge brokering in biosecurity: How international linkages and learnings can help us build a better system	2017	Lye, J.
148	Zero-tolerance biosecurity protects high-conservation-value island nature reserve	2017	Scott, J. K. and McKirdy, S. J. and van der Merwe, J. and Green, R. and Burbidge, A. A. and Pickles, G. and Hardie, D. C. and Morris, K. and Kendrick, P. G. and Thomas, M. L. and Horton, K. L. and O'Connor, S. M. and Downs, J. and Stoklosa, R. and Lagdon, R. and Marks, B. and Nairn, M. and Mengersen, K.
149	Helping local industries help themselves in a multi-level biosecurity world - Dealing with the impact of horticultural pests in the trade arena	2017	Kruger, H.
150	Prioritizing plant eradication targets by re-framing the project prioritization protocol (PPP) for use in biosecurity applications	2017	Dodd, A. J. and Ainsworth, N. and Hauser, C. E. and Burgman, M. A. and McCarthy, M. A.
151	Early warning system in Tunisia: evolution, challenges and role of laboratory, biosecurity, environment and modeling of infectious diseases	2017	Alaya, Nissaf Bouafif Ben and Bellali, HÃ©dia and Nachtnebel, Matthias and Hollenweger, Lilian and KÃ¼cÃ¼kali, Dilan and Allani, Riadh
152	Biosecurity 2025: Direction Statement for New Zealands Biosecurity System	2016	Ministry for Primary Industries
153	The role and challenges of new diagnostic technology in plant biosecurity	2016	Mumford, R. A. and Macarthur, R. and Boonham, N.
154	Biosecurity and food security-effective mechanisms for public-private partnerships	2016	Fraser, G.

155	DNA barcoding for biosecurity: case studies from the UK plant protection program	2016	Hodgetts, J. and OstojÃ¡-Starzewski, J. C. and Prior, T. and Lawson, R. and Hall, J. and Boonham, N.
156	Market instruments, biosecurity and place-based understandings of animal disease	2016	Enticott, G.
157	Field application of a combined pig and poultry market chain and risk pathway analysis within the Pacific Islands region as a tool for targeted disease surveillance and biosecurity	2016	Brioudes, A. and Gummow, B.
158	Trade liberalisation and Australian biosecurity: opportunities and challenges under the â€˜shared responsibilityâ€™approach	2016	Richards, Carol and Higgins, Vaughan
159	Acanthaster planci invasions: applying biosecurity practices to manage a native boom and bust coral pest in Australia	2016	Hoey, J. and Campbell, M. L. and Hewitt, C. L. and Gould, B. and Bird, R.
160	Ornamental Fish Importation-Australias New Approach to Managing Biosecurity Risks	2016	Hood, Yuko and Perera, Ramesh Poshitha
161	Treasure Islands: biosecurity in the Hauraki Gulf Marine Park	2016	Bassett, I. E. and Cook, J. and Buchanan, F. and Russell, J. C.
162	The biosecurity approach. A review and evaluation of its application by FAO, internationally and in various countries.	2016	FAO
163	How social and citizen science help challenge the limits of the biosecurity approach: the case of ash dieback	2015	Tsouvalis, Judith
164	Bayesian Estimation for Diagnostic Testing of Biosecurity Risk Material in the Absence of a Gold Standard when Test Data are Incomplete	2015	Clarke, S. J. and Jones, S. A.
165	Improving Smallholder Farmer Biosecurity in the Mekong Region Through Change Management	2015	Young, J. R. and Evans-Kocinski, S. and Bush, R. D. and Windsor, P. A.
166	Risk assessment as a tool for improving external biosecurity at farm level	2015	Lewerin, S. S. and Osterberg, J. and Alenius, S. and Elvander, M. and Fellstrom, C. and Traven, M. and Wallgren, P. and Waller, K. P. and Jacobson, M.
167	An ecohealth assessment of poultry production clusters (PPCs) for the livelihood and biosecurity improvement of small poultry producers in Asia	2015	Wang, L. B. and Basuno, E. and Nguyen, T. and Aengwanich, W. and Ilham, N. and Li, X. Y.
168	Biosecurity and the multiplication of crises in the Egyptian agri-food industry	2015	Dixon, M. W.
169	The Marine Biosecurity Portholeâ€“a web-based information system on non-indigenous marine species in New Zealand	2015	Seaward, Kimberley and Acosta, Hernando and Inglis, Graeme J and Wood, Brent and Riding, Timothy AC and Wilkens, Serena and Gould, Brendan

170	A risk based approach to non-native species management and biosecurity planning	2015	Shucksmith, Rachel J and Shelmerdine, Richard L
171	A standardized approach for meeting national and international aquaculture biosecurity requirements for preventing, controlling, and eradicating infectious diseases	2015	Palić, Dušan; Scarfe, A David; Walster, Christopher I;
172	Diseases of livestock in the Pacific Islands region: Setting priorities for food animal biosecurity	2015	Brioudes, A. and Warner, J. and Hedlefs, R. and Gummow, B.
173	Biosecurity and disease management in China's animal agriculture sector	2015	Wei, X. J. and Lin, W. L. and Hennessy, D. A.
174	The Australian quarantine and biosecurity legislation: Constitutionality and critique	2015	Gray, A.
175	Is Australia's national biosecurity system and the underpinning Intergovernmental Agreement on Biosecurity fit for the future? Discussion Paper	2015	Craik, Wendy; Palmer, David; Sheldrake, Richard;
176	Autonomous surveillance for biosecurity	2015	Jurdak, R. and Elfes, A. and Kusy, B. and Tews, A. and Hu, W. and Hernandez, E. and Kottege, N. and Sikka, P.
177	New Zealand's" Emerging Risks System" for biosecurity	2015	Clark, S and Newfield, M and Reed, C
178	Keeping 'one step ahead' of invasive species: using an integrated framework to screen and target species for detailed biosecurity risk assessment	2015	Singh, S. K. and Ash, G. J. and Hodda, M.
179	Biosecurity in the beef herd	2014	Sibley, D.
180	A multidisciplinary policy approach to food and agricultural biosecurity and defense	2014	McClaskey, Jackie M
181	An online risk-based biosecurity scoring system for pig farms	2014	Dewulf, J
182	Global program for Avian Influenza control and human pandemic preparedness and response: project accomplishments	2014	Jonas, Olga and Warford, Lucas
183	Intervention to improve biosecurity system of poultry production clusters (PPCs) in Thailand	2014	Aengwanich, Worapol and Boonsorn, Thongchai and Srikot, Prayat
184	Biocheck.UGent: a quantitative tool to measure biosecurity at broiler farms and the relationship with technical performances and antimicrobial use	2014	Gelaude, P. and Schlepers, M. and Verlinden, M. and Laanen, M. and Dewulf, J.
185	Biosecurity system in Malaysian fisheries: Gearing up for safe and quality seafood	2014	Raja Sekaran, Hemalatha
186	Biosecurity Status of Food and Agriculture in Nepal	2014	FAO
187	Balancing bioenergy and biosecurity policies: estimating current and future climate suitability patterns for a bioenergy crop	2014	Kriticos, D. J. and Murphy, H. T. and Jovanovic, T. and Taylor, J. and Herr, A. and Raison, J. and O'Connell, D.

188	Plant biosecurity policy-making modelled on the human immune system: What would it look like?	2014	Cook, D. C. and Kristensen, N. P. and Liu, S. and Paini, D. R. and Kerr, P. J. and Sheppard, A. W. and Lonsdale, W. M. and McAllister, R. R. J. and De Barro, P. J.
189	A community-based education trial to improve backyard poultry biosecurity in rural Cambodia	2013	Conan, A. and Ponsich, A. and Goutard, F. L. and Khiev, R. and Tarantola, A. and Sorn, S. and Vong, S.
190	Popular Backyard Flock program reduces biosecurity risks of amateur production	2013	Stinson, S. and Mete, A.
191	Something in the water: biosecurity monitoring of ornamental fish imports using environmental DNA	2013	Collins, R. A. and Armstrong, K. F. and Holyoake, A. J. and Keeling, S.
192	Using internet intelligence to manage biosecurity risks: a case study for aquatic animal health	2013	Lyon, A. and Grossel, G. and Burgman, M. and Nunn, M.
193	On the efficacy of current biosecurity measures at EU borders to prevent the transfer of zoonotic and livestock diseases by travellers	2013	Noordhuizen, J. and Surborg, H. and Smulders, F. J. M.
194	A method for designing complex biosecurity surveillance systems: detecting non-indigenous species of invertebrates on Barrow Island	2013	Whittle, P. J. L. and Stoklosa, R. and Barrett, S. and Jarrad, F. C. and Majer, J. D. and Martin, P. A. J. and Mengersen, K.
195	Extension Education Symposium: The future of biosecurity and antimicrobial use in livestock production in the United States and the role of extension	2012	Clark, S. and Daly, R. and Jordan, E. and Lee, J. and Mathew, A. and Ebner, P.
196	Creating Situational Awareness: A Systems Approach. Baltimore, MD: Center for Biosecurity of UPMC; 2009	2012	Toner, ES
197	Thomson Aberfeldy Demonstration Siteâ€“a strategic weed project. Implementing Victoriaâ€™s biosecurity approach to pest management on public land	2012	Gillespie, Penny and James, Rebecca and Lambourne, Sally
198	Barcoding and border biosecurity: identifying cyprinid fishes in the aquarium trade	2012	Collins, R. A. and Armstrong, K. F. and Meier, R. and Yi, Y. and Brown, S. D. and Cruickshank, R. H. and Keeling, S. and Johnston, C.
199	Improvement in Smallholder Farmer Knowledge of Cattle Production, Health and Biosecurity in Southern Cambodia between 2008 and 2010	2012	Nampanya, S. and Suon, S. and Rast, L. and Windsor, P. A.
200	Use of stakeholder analysis to inform risk communication and extension strategies for improved biosecurity amongst small-scale pig producers	2012	Hernandez-Jover, M. and Gilmour, J. and Schembri, N. and Sysak, T. and Holyoake, P. K. and Beilin, R. and Toribio, Jalml
201	Marine biosecurity: the importance of awareness, support and cooperation in managing a successful incursion response	2012	Piola, R. F. and McDonald, J. I.

202	Biosecurity against invasive alien insect pests: A case study of Chilo sacchariphagus (Lepidoptera: Crambidae) in the southern African region	2012	Way, M. J. and Conlong, D. E. and Rutherford, R. S.
203	GMP+ FSA scheme as part of the integrated system for providing biosecurity in animal production	2012	Žikić, D; Ušćebrka, G; Stojanović, S; Kanački, Z;
204	'Getting the biosecurity architecture right' in the Asia-Pacific region	2012	Minehata, M.
205	People, pathogens and our planet: the economics of one health	2012	World Bank
206	From biodefence to biosecurity: the Obama administration's strategy for countering biological threats	2012	Koblentz, G. D.
207	Governing biosecurity in a neoliberal world: comparative perspectives from Australia and the United Kingdom	2012	Maye, D. and Dibden, J. and Higgins, V. and Potter, C.
208	The Quarantine Chain: Establishing an Effective Biosecurity System to Prevent the Introduction of Invasive Species Into the Galapagos Islands	2012	Bigue, Marcel
209	Reform of Australia's Biosecurity System: New Biosecurity Legislation	2012	Biosecurity, DAFF
210	Comparison of web-based biosecurity intelligence systems: BioCaster, EpiSPIDER and HealthMap	2012	Lyon, A. and Nunn, M. and Grossel, G. and Burgman, M.
211	Biosecurity Incident Management System V1. 0, 29 October 2012	2012	Group, Biosecurity Emergency Preparedness Working
212	Biosecurity Policy and the Use of Geospatial Predictive Tools to Address Invasive Plants: Updating the Risk Analysis Toolbox	2012	Lindgren, C. J.
213	Biosecurity of kiwifruit plants: effects of internal microchip implants on vines for monitoring plant health status	2012	Luvisi, A. and Panattoni, A. and Bandinelli, R. and Rinaldelli, E. and Pagano, M. and Triolo, E.
214	Integrating surveillance and biosecurity activities to achieve efficiencies in national avian influenza programs	2011	Bunn, D. and Beltran-Alcrudo, D. and Cardona, C.
215	Embryo transfer: a comparative biosecurity advantage in international movements of germplasm	2011	Thibier, M.
216	Biosecurity and vaccination strategies to minimise the effect of an equine influenza outbreak on racing and breeding	2011	Arthur, R. J. and Suann, C. J.
217	Quantification of biosecurity status in pig herds using an online scoring system	2011	Laanen, M and Ribbens, S and Maes, D and Dewulf, J and KÃ¶fer, J.; Schobesberger, H.
218	A review of international, regional and national biosecurity risk assessment frameworks	2011	Dahlstrom, A. and Hewitt, C. L. and Campbell, M. L.
219	International and national biosecurity strategies in aquatic animal health	2011	Oidtmann, B. C. and Thrush, M. A. and Denham, K. L. and Peeler, E. J.

220	Using a Community Approach to Foster Effective Biosecurity Practices Across Social Borders	2011	Royce, Paul
221		2011	FAO
222	The environmental and biosecurity characteristics of livestock carcass disposal methods: A review	2011	Gwyther, C. L. and Williams, A. P. and Golyshin, P. N. and Edwards-Jones, G. and Jones, D. L.
223	Harmonising the Governance of Farming Risks: agricultural biosecurity and biotechnology in Australia	2011	Dibden, J. and Higgins, V. and Cocklin, C.
224	Biosecurity, risk and policy: a New Zealand perspective	2011	Goldson, S. L.
225	Implementing Information System Innovations to Manage Biosecurity Issues in Australian Food NetChains	2011	Storer, Christine and Noonan, John and Murray-Prior, Roy and Batt, Peter
226	Attitudes towards biosecurity practices relevant to Johne's disease control on beef cattle farms	2010	Benjamin, L. A. and Fosgate, G. T. and Ward, M. P. and Roussel, A. J. and Feagin, R. A. and Schwartz, A. L.
227	Biosecurity in the dairy herd	2010	Sibley, R.
228	Biosecurity on pig herds: Development of an on-line scoring system and the results of the first 99 participating herds	2010	Laanen, M. and Beek, J. and Ribbens, S. and Vangroenweghe, F. and Maes, D. and Dewulf, J.
229	Good practices for biosecurity in the pig sector - Issues and options in developing and transition countries	2010	FAO, OIE, World Bank
230	Implementing poultry vaccination and biosecurity at the village level in Tanzania: a social strategy to promote health in free-range poultry populations	2010	Msoffe, P. L. M. and Bunn, D. and Muhairwa, A. P. and Mtambo, M. M. A. and Mwamhehe, H. and Msago, A. and Mlozi, M. R. S. and Cardona, C. J.
231	UAE poultry farm premises ID and biosecurity	2010	Fadel, M. A. and Hussein, A. S.
232	Xylella fastidiosa: a model for analyzing agricultural biosecurity	2010	Ancona, V. and Appel, D. N. and de Figueiredo, P.
233	Towards a Global Barcode Library for Lymantria (Lepidoptera: Lymantriinae) Tussock Moths of Biosecurity Concern	2010	deWaard, J. R. and Mitchell, A. and Keena, M. A. and Gopurenko, D. and Boykin, L. M. and Armstrong, K. F. and Pogue, M. G. and Lima, J. and Floyd, R. and Hanner, R. H. and Humble, L. M.
234	Prioritizing Biosecurity Risks Using a Participatory Decision-Making Tool	2010	Hurley, M. V. and Lowell, K. E. and Cook, D. C. and Liu, S. A. and Siddique, A. B. and Diggle, A.
235	Biosecurity, Expertise and the Institutional Void: The Case of Bovine Tuberculosis	2009	Enticott, G. and Franklin, A.
236	Successful spread of a biocontrol agent reveals a biosecurity failure: elucidating long distance invasion pathways for Gonatocerus ashmeadi in French Polynesia	2009	Petit, J. N. and Hoddle, M. S. and Grandgirard, J. and Roderick, G. K. and Davies, N.
237	Government as biosecurity communicator: the 2006 spinach advisory	2009	Chess, C. and Reilly, M. A. and Cuite, C.

238	The role of plant biosecurity in preventing and controlling emerging plant virus disease epidemics	2009	Rodoni, B.
239	Biosecurity and risk management for dairy replacements	2008	Maunsell, F. and Donovan, G. A.
240	Biosecurity after the event: risk politics and animal disease	2008	Donaldson, A.
241	Biosecurity for Highly Pathogenic Avian Influenza	2008	FAO, OIE, World Bank
242	Development of the shrimp industry in the Western Indian Ocean-a holistic approach of vertical integration, from domestication and biosecurity to product certification	2008	Le Groumelec, Marc and Rigolet, Vincent and Duraisamy, Panchayuthapani and Vandeputte, Marc and Rao, Vemulapali Manavendra
243	National biosecurity approaches, plans and programmes in response to diseases in farmed aquatic animals: evolution, effectiveness and the way forward	2008	Hastein, I. and Binde, M. and Hine, M. and Johnsen, S. and Lillehaug, A. and Olesen, N. J. and Purvis, N. and Scarfe, A. D. and Wright, B.
244	Economic aspects of agricultural and food biosecurity	2008	Hennessy, D. A.
245	Testing island biosecurity systems for invasive rats	2008	Russell, J. C. and Beaven, B. M. and MacKay, J. W. B. and Towns, D. R. and Clout, M. N.
246	Use of data mining techniques to investigate disease risk classification as a proxy for compromised biosecurity of cattle herds in Wales	2008	Ortiz-Pelaez, A. and Pfeiffer, D. U.
247	Flexible boundaries in biosecurity: accommodating gorse in Aotearoa New Zealand	2008	Barker, K.
248	Signals come and go: syndromic surveillance and styles of biosecurity	2008	Fearnley, L.
249	Biosafety and biosecurity: Applicabilities of the biological security	2008	Cardoso, Tado and Navarro, Mbmda and Soares, B. E. C. and Tapajos, A. M.
250	The practice of biosecurity in Canada: public health legal preparedness and Toronto's SARS crisis	2008	Van Wagner, E.
251	Biosecurity: Educational programs	2007	Bradley, F. A.
252	Mechanisms for the prevention of marine bioinvasions for better biosecurity	2007	Hewitt, C. L. and Campbell, M. L.
253	Development of an analytical tool to assess Biosecurity legislation	2007	Manzella, Daniele and Vapnek, Jessica
254	Advances in biosecurity to 2010 and beyond: towards integrated detection, analysis and response to exotic pest invasions	2007	French, N. P. and Gemmell, N. J. and Buddle, B. M.
255	Enhancing control of highly pathogenic avian influenza in developing countries through compensation: issues and good practice	2006	Hristov, Manush A and Bank, World
256	Canadas approach to aquatic animal biosecurity: experience and evolution	2006	McGladdery, Sharon E and Zurbrigg, Richard E

257	Allocating Vote: Biosecurity-towards an economics-based approach for setting priorities for the importation of goods	2006	Ryan, M
258	Biosecurity Awareness and Peri-Urban Landholders: a case study approach	2005	Aslin, Heather J and Mazur, N
259	An integrated approach to biosecurity on UK cattle and sheep farms; evaluating existing measures for endemic diseases against exotic threatsâ€“Extensionâ€™	2004	DEFRA
260	New Zealand marine biosecurity: delivering outcomes in a fluid environment	2004	Hewitt, C. L. and Willing, J. and Bauckham, A. and Cassidy, A. M. and Cox, C. M. S. and Jones, L. and Wotton, D. M.
261	Using Web-Based Distance Diagnostic and Identification System a Tool for Biosecurity	2003	Xin, Jiannong and Momol, MT and Sprenkel, RK and Beck, Howard W and Zazueta, FS and Vergot, P and Wisler, GC
262	Symposium: Agro-terrorism: Biological threats and biosecurity measures - Food security issues - A potential comprehensive plan	2003	Norton, R. A.
263	MiTAP for biosecurity - A case study	2002	Damianos, L. and Ponte, J. and Wohlever, S. and Reeder, F. and Day, D. and Wilson, G. and Hirschman, L.
264	Biosecurity: Moving toward a comprehensive approach	2002	Meyerson, L. A. and Reaser, J. K.
265	Dealing with the human dimensions of invasive alien species within New Zealandâ€™s biosecurity system	2001	Warren, Paula
266	A weed risk assessment model for use as a biosecurity tool evaluating plant introductions	1999	Pheloung, P. C. and Williams, P. A. and Halloy, S. R.
